# Functional and structural analysis of AT-specific minor groove binders that disrupt DNA–protein interactions and cause disintegration of the *Trypanosoma brucei* kinetoplast

**DOI:** 10.1093/nar/gkx521

**Published:** 2017-06-16

**Authors:** Cinthia R. Millan, Francisco J. Acosta-Reyes, Laura Lagartera, Godwin U. Ebiloma, Leandro Lemgruber, J. Jonathan Nué Martínez, Núria Saperas, Christophe Dardonville, Harry P. de Koning, J. Lourdes Campos

**Affiliations:** 1 Departament d’Enginyeria Química, EEBE, Universitat Politècnica de Catalunya, 08019 Barcelona, Spain; 2 Instituto de Química Médica, IQM–CSIC, 28006 Madrid, Spain; 3 Institute of Infection, Immunity and Inflammation, College of Medical, Veterinary and Life Sciences, University of Glasgow, Glasgow G12 8TA, UK; 4 The Wellcome Centre for Molecular Parasitology, Institute of Infection, Immunity and Inflammation, University of Glasgow, Glasgow G12 8TA, UK

## Abstract

*Trypanosoma brucei*, the causative agent of sleeping sickness (Human African Trypanosomiasis, HAT), contains a kinetoplast with the mitochondrial DNA (kDNA), comprising of >70% AT base pairs. This has prompted studies of drugs interacting with AT-rich DNA, such as the *N*-phenylbenzamide bis(2-aminoimidazoline) derivatives **1** [4-((4,5-dihydro-1*H*-imidazol-2-yl)amino)-*N*-(4-((4,5-dihydro-1*H*-imidazol-2-yl)amino)phenyl)benzamide dihydrochloride] and **2** [*N*-(3-chloro-4-((4,5-dihydro-1*H*-imidazol-2-yl)amino)phenyl)-4-((4,5-dihydro-1*H*-imidazol-2-yl)amino)benzamide] as potential drugs for HAT. Both compounds show *in vitro* effects against *T. brucei* and *in vivo* curative activity in a mouse model of HAT. The main objective was to identify their cellular target inside the parasite. We were able to demonstrate that the compounds have a clear effect on the S-phase of *T. brucei* cell cycle by inflicting specific damage on the kinetoplast. Surface plasmon resonance (SPR)–biosensor experiments show that the drug can displace HMG box-containing proteins essential for kDNA function from their kDNA binding sites. The crystal structure of the complex of the oligonucleotide d[AAATTT]_2_ with compound **1** solved at 1.25 Å (PDB-ID**: 5LIT**) shows that the drug covers the minor groove of DNA, displaces bound water and interacts with neighbouring DNA molecules as a cross-linking agent. We conclude that **1** and **2** are powerful trypanocides that act directly on the kinetoplast, a structure unique to the order Kinetoplastida.

## INTRODUCTION

**Figure 1.**
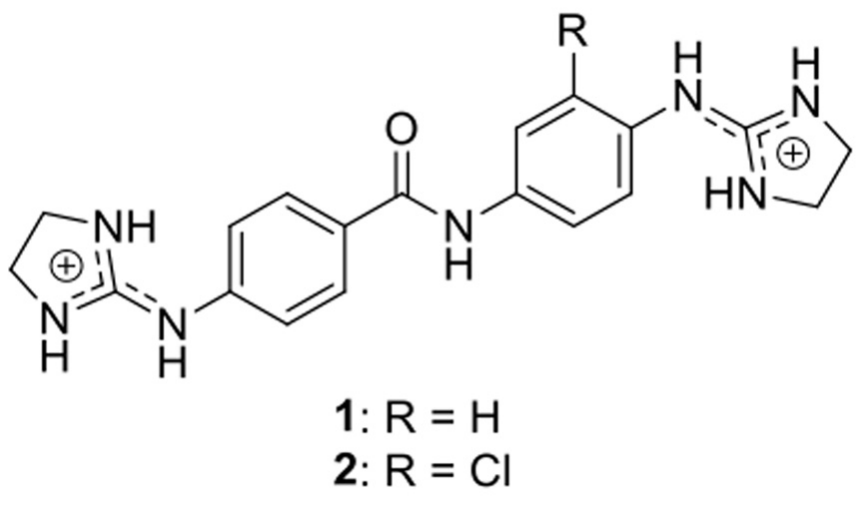
Structure of compounds **1** and **2**.

Human African trypanosomiasis (HAT), also known as sleeping sickness, is a neglected tropical disease that is almost invariably fatal if left untreated. It is caused by subspecies of the protozoan parasite *Trypanosoma brucei* which is transmitted to humans by the tsetse fly vector. Approximately 55 million people distributed over a surface of 340 000 km^2^ in 33 sub-Saharan Africa countries are estimated to be at different levels of risk of contracting sleeping sickness (1). Most of the affected populations live in remote rural areas with limited access to health services, which complicates the diagnosis and treatment of cases in Africa's poorest countries. The current drugs used to treat HAT (suramin, pentamidine, melarsoprol and nifurtimox-eflornithine combination therapy, or NECT) are toxic and sometime ineffective due to the appearance of drug-resistant strains of *T. brucei* (2). In the last decades, efforts have been made to discover improved drugs to treat HAT (1). In previous reports, we have shown that compound **1**, 4-((4,5-dihydro-1*H*-imidazol-2-yl)amino)-*N*-(4-((4,5-dihydro-1*H*-imidazol-2-yl)amino)phenyl)benzamide dihydrochloride, and a series of chloro, fluoro and pyridinyl derivatives, displayed excellent antitrypanosomal activity *in vitro* and were selective toward *Trypanosoma brucei* (3). Compound **1** was curative by oral administration in a mouse model of acute *T. b. rhodesiense* infection (4) demonstrating a great potential as chemotherapeutic agent. Compound **2**, a chloro analogue closely related to **1**, was recently shown to be more potent than **1***in vitro* (3) (Figure 1).

Previous studies by our group (5–10) and by others (11–15) have shown that bis(2-aminoimidazoline) drugs of this class are DNA minor groove binders with excellent affinity and selectivity toward AT-rich DNA regions. Since transcription and activity of other DNA-dependent enzymes may all be inhibited by DNA binders (16,17), this interaction is thought to be primarily responsible for the antitrypanosomal activity of this class of compounds. In fact, the dicationic nature of these molecules is the driving force that compels them to accumulate in the charged mitochondrion of the parasite (i.e. negative inside); the high local concentration aids their anti-parasite effects by binding to DNA or mitochondrial proteins, as shown for other trypanocidal cations (18–21).

For many years, our group has focused on a series of bis(2-aminoimidazolines) and their interactions with the minor groove of DNA, almost exclusively with adenine and thymine (AT) sequences. Previous studies have shown the 3D structure of minor groove-binding drugs in complex with AT-rich oligonucleotides, which interact with neighbouring DNA molecules, e.g. pentamidine in complex with AT alternating sequences (22) and CD27 with A-tracks (8).

Here, we report a detailed study of the interaction of two new drugs, the bis(2-aminoimidazolinium) compounds **1** and **2**, with AT-rich DNA sequences. Since kinetoplast DNA has a high content of AT-rich sequences, we aimed to discover whether parasite DNA was a target for these compounds inside the cell. We evaluated the *in vitro* and *in vivo* effect of these drugs against bloodstream form *T. brucei* and, by a combination of flow cytometry and imaging techniques, confirmed their cellular target as kDNA.

We also analysed the kDNA genome and found it abundant in AAA or TTT sequences (see Supplementary Figure S1, available as Supplementary data), which could serve as possible binding sites for the drugs. The X-ray crystal structure of this DNA sequence in complex with compound **1** gave us a model for the interaction at atomic resolution of 1.25 Å. In support of drug action through direct binding to kDNA we demonstrated that compound **2** displaces the human proteins HMGA1a and HMGB1 from DNA. This result is of particular interest to understand its trypanocidal activity because the mitochondrial HMG-box protein TbKAP6, which is similar to HMGB1, is essential for kDNA replication and maintenance (23).

## MATERIALS AND METHODS

### Strains and cultures

All parasite cultures used in this study were *Trypanosoma brucei brucei* bloodstream forms (BF) of strain Lister 427/MiTat1.2 (wild-type, Tb427WT) or of *T. b. brucei* ISMR1. Strain ISMR1 is a clonal, Tb427WT-derived dys-kinetoplastid cell line, adapted to *in vitro* growth in 1 μM of isometamidium (ISM) by stepwise increases in the medium concentration of the drug (19). All strains were cultured under standard conditions (37°C, 5% CO_2_) in HMI-9 medium (Gibco, UK) supplemented with 2 mM β-mercaptoethanol (Sigma-Aldrich) and 10% fetal bovine serum (FBS; Gibco) exactly as described (24).

### Drug sensitivity assays

Fifty percent effective concentrations (EC_50_) were determined using the fluorescence viability indicator dye Alamar Blue (resazurin sodium salt, Sigma-Aldrich) as described (25), with small modifications. Resazurin is metabolized by live cells to resorufin and the fluorescent signal generated is thus proportionate to the number of live cells (25). Thus, the assay measures cell numbers rather than detecting cell lysis, and low cell numbers can be the result of growth inhibition/ cell cycle arrest or of a lethal effect of the test compound on part of the cell population. Doubling dilutions of test compounds were prepared over two rows of a 96-well plate, leaving the last well drug-free as a control, before the addition of an equal volume of cell suspension to each well, to get a final cell density of 1 × 10^4^ cells/ml. The plates were incubated at 37°C/5% CO_2_ for 72 h after which 20 μl of resazurin solution (125 μg/ml in phosphate-buffered saline, pH 7.4) was added followed by another incubation for 24 h. Fluorescence was determined using a FLUOstar Optima plate reader (BMG Labtech, Durham, NC, USA), at excitation and emission wavelengths of 530 and 590 nm, respectively. Data were analysed and plotted to a sigmoid curve with variable slope using Prism 5.0 (GraphPad, San Diego, CA, USA).

### Effect of bis(2-aminoimidazolinium) *N*-phenylbenzamide compounds on growth of BF *T. brucei*

The effects of the bis(2-aminoimidazolinium) compounds **1** and **2** on growth of BF *Trypanosoma brucei brucei* strain Lister 427 were investigated by incubating cultures under standard conditions (HMI-9/10% FBS; 37°C, 5% CO_2_) in the presence or absence of 0.5, 1, 2.5 and 5 times their EC_50_ values for up to 48 h. Cells were seeded at a density of 2 × 10^4^ cells/ml using a haemocytometer. Samples were taken in triplicate at the following times (h) after initiation of the experiment: 0, 4, 8, 24, 36, 48.

### Assessment of cell cycle progression in *T. brucei*

#### Fluorescence microscopy

Nuclei and kinetoplasts were visualized by using the fluorescent dye 4’,6-diamidino-2-phenylindole (DAPI) on BF trypanosomes after fixation. 50 μl of cells at 2 × 10^6^ cells/ml were spread onto a glass microscope slide, left to dry in a fume hood and fixed in 4% paraformaldehyde in fresh PBS for 10 min at room temperature. The slides were washed with 1 × PBS. The PBS was gently removed by inclining the slide on a soft tissue and a drop of Vectashied^®^ mounting medium containing DAPI dihydrochloride (Vector Laboratories, USA) was placed on the slide before covering with a cover slip. The slide was then viewed under UV light on a Zeiss Axioskop 2 fluorescent microscope (Carl Zeiss Microscopy, USA). Five hundred cells were recorded for each sample, and scored for DNA configuration following these groups: 1N1K, 1N2K, 2N2K (Early) and 2N2K (Late) (N, nucleus; K, kinetoplast). The effect of test compounds on DNA configuration was determined at 0, 8 and 24 h; untreated cultures served as control.

#### Flow cytometry

DNA content of BF trypanosomes was measured by flow cytometry using the fluorescent dye propidium iodide (PI), as described (18,26). Samples were analysed using a BD FACSCalibur™ system (BD Biosciences). Data was acquired from the FSC, SSC, FL2- W and FL2-A detectors using BD CellQuest™ software (BD Biosciences), and further analysed and graphically represented using the ©FlowJo 10.2 software.

### Mitochondrial membrane potential

The mitochondrial membrane potential of treated and untreated cells was assessed by using tetramethylrhodamine ethyl ester (TMRE) following a protocol previously described (18). Samples were analysed using a BD FACSCalibur™ system (BD Biosciences). Data were acquired from the FL2-height detector using BD CellQuest™ software (BD Biosciences), and further analyzed and graphically represented using the ©FlowJo software, version 10.2. Valinomycin (100 nM) and troglitazone (10 μM) were employed as negative (mitochondrial membrane depolarisation) and positive (mitochondrial membrane hyperpolarisation) controls, respectively (27). Mitochondrial membrane potential was determined at 0, 3, 6 and 12 h. The detector was calibrated so that the peak of control cells (Tb427WT not exposed to any test compound) was set at 100 arbitrary units, i.e. with 50% of cells at >100 A.U. and 50% at <100 A.U. The data are presented as percent of the cell population with a fluorescence >100 A.U.

### Cellular localization of the compound in the BF *T. brucei*

The localization and distribution of the fluorescent compound **2** in the BF trypanosomes was studied by fluorescence microscopy. Trypanosome cultures were grown in HMI-9/FCS media, cell density was adjusted to 2 × 10^6^ cells/ml and the compound **2** was added to make a concentration of 5 μM; a negative control (drug free sample) was also prepared. 1 ml of sample was taken every time point and transferred into an empty tube, and 1 μl of 1 mM solution of MitoTracker^®^ Red CMXRos (Thermo Fisher Scientific) in DMSO was added to make a final concentration of 100 nM. Samples were incubated at 37°C and 5% CO_2_ for 5 min. Subsequently, samples were washed with filter-sterilised 1× PBS and spun at 1500 × g. Pellets were re-suspended in 50 μl of PBS and spread into a glass microscope slide. The cells were dried in a fume hood and fixed in 4% formaldehyde/PBS fresh for 10 min at room temperature. Cells were permeabilized with cooled methanol for 5–10 min at –20°C, washed once or twice with 1× PBS and then washed twice with Hanks' Balanced Salt Solution (HBSS). Enough solution 1:30 000 (167 nM) of green-fluorescent counterstain SYTOX^®^ Green nuclei acid stain (Thermo Fisher Scientific) in HBSS to cover all the cells was added to the slides and incubated for 15–30 min protected from light. Finally, cells were washed twice with HBSS and the slides were covered with a cover slip. The location of the test compound was determined at 0, 3, 6 and 24 h. The slide was then viewed under a DeltaVision Core microscope (Image Solutions - Applied Precision, United Kingdom) under several fluorochromes (blue for **2**, green for the SYTOX^®^ and red for the Mitotracker^®^), as well as under UV light. Image acquisition was controlled using Zen software (Zeiss) and SoftWorx software was used for further image processing.

### Transmission electron microscopy

Transmission electron microscopy was used to assess the action of the compounds on *T. brucei* ultrastructure. Cells were harvested and the density was adjusted to 2 × 10^6^ cells/ml. The samples incubated 3 and 24 h with 5 μM of the compound, as well as untreated cells for control were centrifuged at 610 × g for 10 min at room temperature and the pellet was washed once with 1× PBS and re-suspended in 1 ml of the fixative solution containing 2.5% of glutaraldehyde and 4% paraformaldehyde in 0.1 M phosphate buffer (pH 7.4) and left at 4°C overnight. Then, samples were centrifuged at 4000 rpm for 8 min and pellet was washed three times with 0.1 M phosphate buffer (pH 7.4). Cells were post-fixed in a solution of 1% osmium tetroxide for 1 h on ice, and washed with the phosphate buffer to remove excess of osmium tetroxide. Then, cells were kept in 0.5% uranyl acetate solution for 30 min, washed three times with distilled water and dehydrated by using a series of increasing concentrations of acetone (30, 50, 70, 90 and 100%). Next, cell infiltration was performed using an epoxy resin. Thin sections (50–60 nm) were observed in a Tecnai T20 (FEI) at 200 kV.

### *In vivo* activity against *T. b. rhodesiense*

Compound **2** was assayed *in vivo* in the STIB900 mouse model of acute infection. The compound was administered to a group of four female NMRI mice by intraperitoneal route using a 4-day treatment regimen of 20 mg/kg/day using a previously reported protocol (9). Parasitemia was monitored over a 60-day observation period. Mice were considered cured when no relapse was detected in the tail blood over the observation period. The *in vivo* efficacy studies were conducted at the Swiss Tropical and Public Health Institute (Basel) according to the rules and regulations for the protection of animal rights of the Swiss ‘Bundesant für Veterinärwesen’. They were approved by the veterinary office of Canton Basel-stadt, Switzerland.

### Crystallization assays

#### Synthesis

The deoxyoligonucleotide d[AAATTT]_2_ was synthesized at the Pasteur Institute as the ammonium salt on an automatic synthesizer by the phosphoramidite method. It was purified by gel filtration and reverse-phase HPLC. The final concentration was calculated from the *A*_260_ using an extinction coefficient of 89.84 mM^−1^ cm^−1^.

The synthesis of 4-((4,5-dihydro-1*H*-imidazol-2-yl)amino)-*N*-(4-((4,5-dihydro-1*H*-imidazol-2-yl)amino)phenyl)benzamide dihydrochloride (**1**) and its chloro derivative (**2**) were performed following a procedure described previously (3,6).

#### Crystallization

The crystal was grown by vapour diffusion at 4°C on hanging-drops. We used the following conditions: a pre-incubated DNA-compound **1** complex in sodium cacodylate buffer pH 6.0 was added to the drop with final concentrations of 0.2 mM DNA duplex, 0.4 mM compound **1**, 25 mM sodium cacodylate buffer pH 6.0, 10 mM magnesium acetate (Sigma), 0.1 mM spermine (Fluka) and 5% 2-methyl-2,4-pentanediol (MPD; Sigma-Aldrich); equilibrated against a 20% MPD reservoir. MPD acts both as a precipitant and a cryoprotectant. The concentration of the reservoir was increased gradually up to 50% MPD. A single crystal appeared after 14 months.

#### Data collection and structure determination

The crystal was flash-frozen and stored in liquid Nitrogen. A PILATUS 6M detector on beamline BL13-XALOC (28) at the ALBA synchrotron, Barcelona, Spain was used for data collection at 100 K and a wavelength of 0.979 Å, with a maximum resolution of 1.2 Å. The data were integrated using *iMOSFLM* (29) and scaled with *Aimless Pointless* (30) from the *CCP4* suite (31). The space group turned out to be the monoclinic I121. A theoretical B-DNA model was constructed using *TURBO-FRODO* (http://www.afmb.univ-mrs.fr/-TURBO-), with a base-pair stacking of 3.286 Å, a uniform twist of 36° and Watson–Crick base-pair pairing. It served as a starting search model for molecular replacement with *MOLREP* (32). Due to the lack of success after an extensive search in different monoclinic space groups, we generated another theoretical model formed by half of the oligonucleotide and placed it in the correct position by molecular replacement using *Phaser* (33). The missing base pairs of the oligonucleotide were added with *Coot* (34) following the well-defined electron diffraction map. The replacement was next refined with *REFMAC5* (35) and real-space refinement was performed with *Coot*. In this way we placed one duplex and two single strands of DNA in the asymmetric unit. The coordinates and stereochemical restraint dictionary of the compound **1** molecule were generated with *Phenix* (36), and one drug per duplex was placed in the minor groove of the DNA structure using *Coot*. One DNA duplex and two DNA single strands with three compound **1** molecules formed the final model. Several cycles of maximum-likelihood isotropic restrained refinement were performed with *REFMAC5* to 1.247 Å resolution. Finally, a last round of refinement with anisotropic restrictions was completed to obtain the final values of R_work_ = 0.1205 and R_free_ = 0.1668 in a resolution range of 36.137–1.247 Å, with a completeness of 92.54%. Four magnesium ions were detected. Solution coordinates have been deposited in the Protein Data Bank as PDB-ID: **5LIT**. The DNA structural parameters were calculated using the *3DNA* software (37). Drawings were prepared with *PyMOL* (http://www.pymol.org) and *CCP4mg* (38).

### Surface plasmon resonance (SPR)–biosensor experiments

The 5’-biotin labelled DNA hairpins were purchased from Sigma-Aldrich with reverse-phase HPLC purification. The DNA hairpin sequences included 5’-biotin-GGGAATAATCGCGATTATTCCCCAATAATCGCGATTATT [AATAAT_ATTATT], 5’-biotin-CGAATTCGTCTCCGAATTCG-3’ [AATT], 5’-biotin-CATATATATCCCCATATATATG-3’ [(AT)_4_], and 5’-biotin-CGCGCGCGTTTTCGCGCGCG-3’ [(CG)_4_]. The oligomers were dissolved in 10 mM HEPES, 150 mM NaCl, pH 7.4.

#### HMGB1 protein expression and purification

Plasmid pGEX2T containing GST-tagged rat HMGB1 box A and box B domains (Lys8–Lys165), kindly provided by Dr M.E.A. Churchill (University of Colorado, USA) (39) (see Supplementary Figure S2), was expressed in *Escherichia coli* Rosetta(DE3)pLysS strain (Novagen). Protein expression and purification was done as in (40).

#### HMGA1a Δ50-91 protein expression and purification

Plasmid pET3a-HMG-IΔ50–91 containing human HMGA1a (amino acids 50–91) was kindly provided by Dr R. Reeves (Washington State University, USA). This fragment contains the AT-hook II and AT-hook III domains of the protein (Supplementary Figure S2). AT-hook domains allow the binding of the protein to the minor groove of AT-rich DNA sequences. HMGA1a Δ50–91 was expressed in *Escherichia coli* Rosetta(DE3)pLysS strain. Cells were grown in LB medium containing ampicillin and chloramphenicol at 37°C with vigorous shaking. When the absorbance at 600 nm reached 0.6, expression was induced for 3 h with 1 mM IPTG. The bacterial cells were harvested by centrifugation at 4000 rpm for 10 min. Bacterial pellets were resuspended in ice-cold 5% HClO_4_/0.5% Triton X-100 and protein was precipitated with 25% TCA as described by Reeves and Nissen (41). Protein was first purified by cation-exchange chromatography using a CM52 (Whatman) column and a linear gradient to a final concentration of 1 M NaCl in 25 mM Tris, pH 7.0. The purest fractions, as assessed by SDS-PAGE, were pooled and concentrated for a final purification via size-exclusion chromatography (Superdex Peptide 10/300 GL, GE Healthcare) in 100 mM NaCl, 100 mM Tris, pH 7.5. Final pure samples were dialysed in 25 mM HEPES, 50 mM NaCl, 1 mM DTT pH 7,4. The final protein concentration was calculated using an extinction coefficient of 38 200 mol^−1^ cm^−1^ at 220 nm (42).

#### SPR-biosensor experiments: binding affinity and binding inhibition

SPR binding and competitive experiments were performed at 25°C with a Biacore X-100 apparatus (Biacore GE). HEPES 1 buffer containing 10 mM HEPES, 3 mM EDTA, 200 mM NaCl, and surfactant P-20 at 0.05% (v/v) pH 7.4, was used for the competition experiments with HMGA1a and HMGB1 proteins, and also for the dilution of HMGA1a and HMGB1. The DNA hairpins were immobilized on a streptavidin-derivatized gold chip (SA chip from Biacore) by injection of a 25 nM hairpin DNA solution with a flow rate of 1 μl/min until ∼400 RU were reached. Flow cell 1 was used as reference while flow cell 2 was immobilized with the hairpins in different chips. Direct binding of HMGA1a and HMGB1 was measured by injection of increasing concentrations of each protein over the immobilized DNA surfaces at a flow rate of 50 μl/min for a period of 60 s followed by a dissociation period of 120 s. Regeneration of the surface was made with NaCl 200 mM/NaOH 10 mM using a flow rate of 10 μl/min during 30 s. The binding affinity was determined by fitting the results to a two sites binding model according to the equation:

\begin{equation*}r = ({K_1}{C_f} + 2{K_1}{K_2}C_f^2)/(1 + {K_1}{C_f} + {K_1}{K_2}C_f^2),\end{equation*}

where *r* is the moles of bound compound per mole of DNA hairpin duplex, *C_f_* is the free concentration at the equilibrium, and *K*_1_ and *K*_2_ the microscopic binding constants.

The competition experiments were prepared with samples containing a fixed concentration of HMGA1a and HMGB1 proteins (2 μM) and a series of concentrations of compound **2** ranging from 0.05 to 200 μM (for HMGA1a) or 400 μM (for HMGB1) in HEPES 1 buffer. The samples were injected to the immobilized DNA surfaces at a flow rate of 50 μl/min for a period of 60 s followed by a dissociation period of 150 s. The regeneration conditions were similar to the binding experiments described above. The binding responses (RU) at steady state were averaged and normalized by setting the RU with the proteins HMGA1a and HMGB1 alone as 100% binding to DNA and the RU with saturation by the inhibitor as 0%. IC_50_ values were determined by fitting the inhibition data with a model according to a competition system with 1:1 binding stoichiometry for HMGA1a and HMGB1 and two site-binding for competitor:

\begin{equation*}\begin{array}{@{}*{1}{l}@{}} {\% {\rm{proteins}}\;{\rm{binding}}\;{\rm{to}}\;{\rm{DNA}}}\\ { = 100/\left[ {1 + {\rm{C}}(1 + {{\rm{K}}_{{\rm{c}}2}}{\rm{C}})/[{\rm{I}}{{\rm{C}}_{50}}(1 + {{\rm{K}}_{{\rm{c}}2}}{\rm{I}}{{\rm{C}}_{50}})]} \right]} \end{array}\end{equation*}

where *K*_c2_ is a macroscopic binding constant for inhibitor binding to DNA, IC_50_ is the concentration of the inhibitor that causes 50% inhibition of HMGA1a and HMGB1 binding to DNA, and *C* is the concentration of inhibitor (43).

## RESULTS

### Functional analysis

#### Resistance profile of isometamidium-adapted cell line ISMR1 and the parental cell line Tb427WT

As the starting hypothesis was that kDNA might be a target for compounds **1** and **2**, they were tested in parallel against the standard *T. brucei* laboratory strain Tb427WT and the isometamidium-adapted cell line ISMR1 (19), which is dyskinetoplastic, meaning that the strain has adapted to the loss of its kinetoplast. *In vitro* drug sensitivity assays established that ISMR1 was highly resistant to ISM when the EC_50_ values of the resistant clone were compared to those of the parent Tb427WT strain (Table 1). Significant cross-resistance is displayed by ISMR1 to the compounds **1** and **2**, with 127- and 132-fold increases in EC_50_ values, respectively, indicating that the independence from kDNA had made the cells resistant to the test compounds.

**Table 1.** Activity of compounds **1** and **2** against *T. brucei* 427WT and the isometamidium-resistant strain ISMR1

Compounds	*T. b. brucei* 427WT	*T. b. brucei* ISMR1	Resistance Factor vs. Tb247WT
	EC_50_ (μM) ± SEM	EC_50_ (μM) ± SEM	
**1**	0.83 ± 0.08^a^	105.3 ± 3.2	127 ***
**2**	0. 220 ± 0.002^a^	29.0 ± 0.7	132 ***
Isometamidium	0.016 ± 0.001	1464 ± 94	92522 ***

EC_50_ values are given as mean of at least three independent determinations and SEM. The resistance factor (RF) is the ratio of the EC_50_ values of the adapted strain and the wild-type control. Statistical significance was determined using a two-tailed unpaired *t*-test.

^a^Reference (3). Asterisks represent *P*-values for statistically significant resistance, as calculated using a two-tailed unpaired Student's *t*-test: ***, *P*-value <0.0001.

#### Compounds **1** and **2** alter the cell cycle of T. brucei

**Figure 2.**
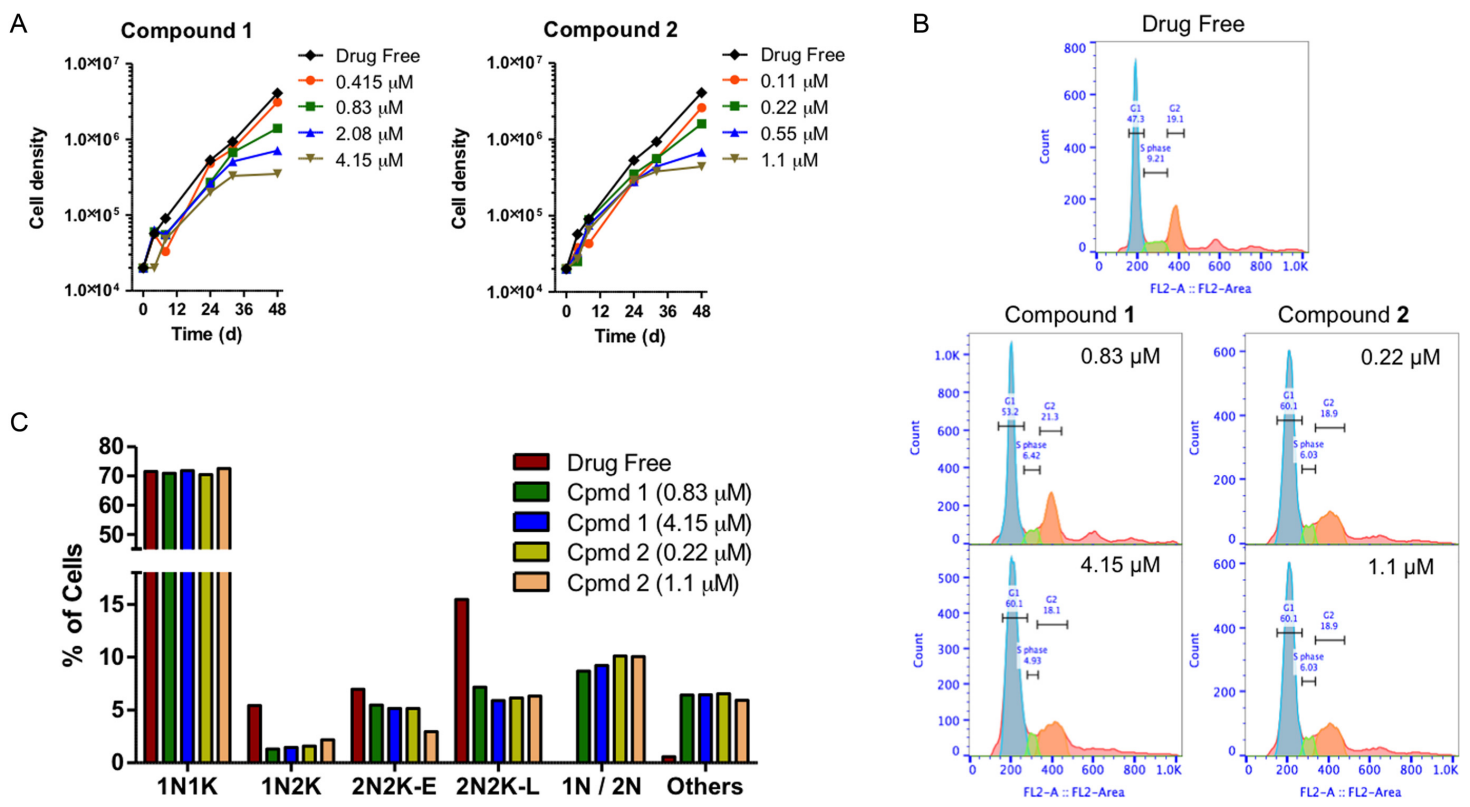
(**A**) Growth curves of untreated control *T. brucei* 427WT and of parallel cultures treated with compounds **1** and **2** at 0.5, 1, 2 and 5 × their EC_50_ values. (**B**) Histograms of flow cytometric analysis of propidium iodide fluorescence associated with Tb427WT trypanosomes. The frames shown show control and exposed (1 × and 5 × EC_50_) to **1** or **2** for 24 h, with most cells in G1 phase (blue peak, single set of chromosomes) and smaller numbers in S-phase (green, DNA synthesis) and G2 phase (orange, 2 sets of chromosomes). (**C**) DNA content of cells treated 24 h with compounds **1** and **2** at 1 × and 5 × EC_50_ as determined by fluorescence microscopy (N, nucleus; K, kinetoplast; 1N/2N, cells with one or two nuclei but no observable kinetoplastid).

In the *T. brucei* cell cycle, replication and division of kDNA necessarily precedes nuclear division (44), and compounds that directly impact on kDNA are thus expected to interfere with cell division. Indeed, both compounds dose-dependently reduced *T. brucei* growth rates and, at concentrations above EC_50_, appeared to induce growth arrest after 24 h (Figure 2A).

To analyze whether compounds **1** and **2** have an effect on the cell cycle, the DNA content was measured by flow cytometry, with emphasis on the ratio between cells with G1 phase of cell cycle and those cells synthesising DNA (S phase of cell cycle), as well as on the appearance of cells in G2 phase. Moreover, in order to observe cell cycle progression in detail, i.e. to visualize DNA configuration from nuclei and kinetoplast, fluorescence microscopy was carried out. The results show a clear effect of the tested compounds on the S phase of the cell cycle after 12 h and an even more remarkable alteration after 24 h of incubation (Figure 2B). The proportion of cells in G2 phases initially showed a slight increase (at 12 h of treatment), and a small reduction later (after 24 h), while an increase of cells in G1 phase was observed. The percentage of treated cells in S phase decreased dose-dependently throughout the experiment, whereas there was no significant change in the S phase for the control which maintained a normal growth rate (see Supplementary Figure S3).

The cellular DNA configuration was assessed using the dye DAPI for compound **1** and SYTOX Green nuclei acid stain for compound **2** (as compound **2** fluoresces at a similar wavelength as DAPI). Cells exposed to the compounds for 8 and 24 h were scored into the following standard groups: 1N1K (one nucleus and one kinetoplast), 1N2K (one nucleus and two kinetoplasts), 2N2K-E (two nuclei and two kinetoplasts, but no clear furrow towards cell division), 2N2K-L (two nuclei and two kinetoplasts when cells were almost completely divided into two daughter cells). Another group was added to the classification: 1N/2N (1 or 2 nuclei and no kinetoplast) due to the high incidence of cells with such a DNA configuration after 8 h (Supplementary Figure S4) or 24 h of treatment (Figure 2C). In cells treated with either compound, there was a dose-dependent reduction in cells with one nucleus and two kinetoplasts, indicative of a failure to initiate a new cell division cycle. Similarly, the number of cells in the early stages of cell division, with two kinetoplasts and nuclei, was approximately halved after 24 h of exposure to the compounds. At the same time, cells without any kinetoplast (1N/2N) started to appear in the treated populations, having apparently concluded mitosis despite the non-division of the kinetoplasts. In addition, completely aberrant cells (‘others’) also appeared in the treated cultures, which typically contained multiple nuclei and/or kinetoplasts, or none at all. The combined evidence shows that **1** and **2** interfere with the normal progression of the *T. brucei* cell cycle, most likely by preventing kinetoplast division.

#### Compounds **1** and **2** cause a slow decline in mitochondrial membrane potential

**Figure 3.**
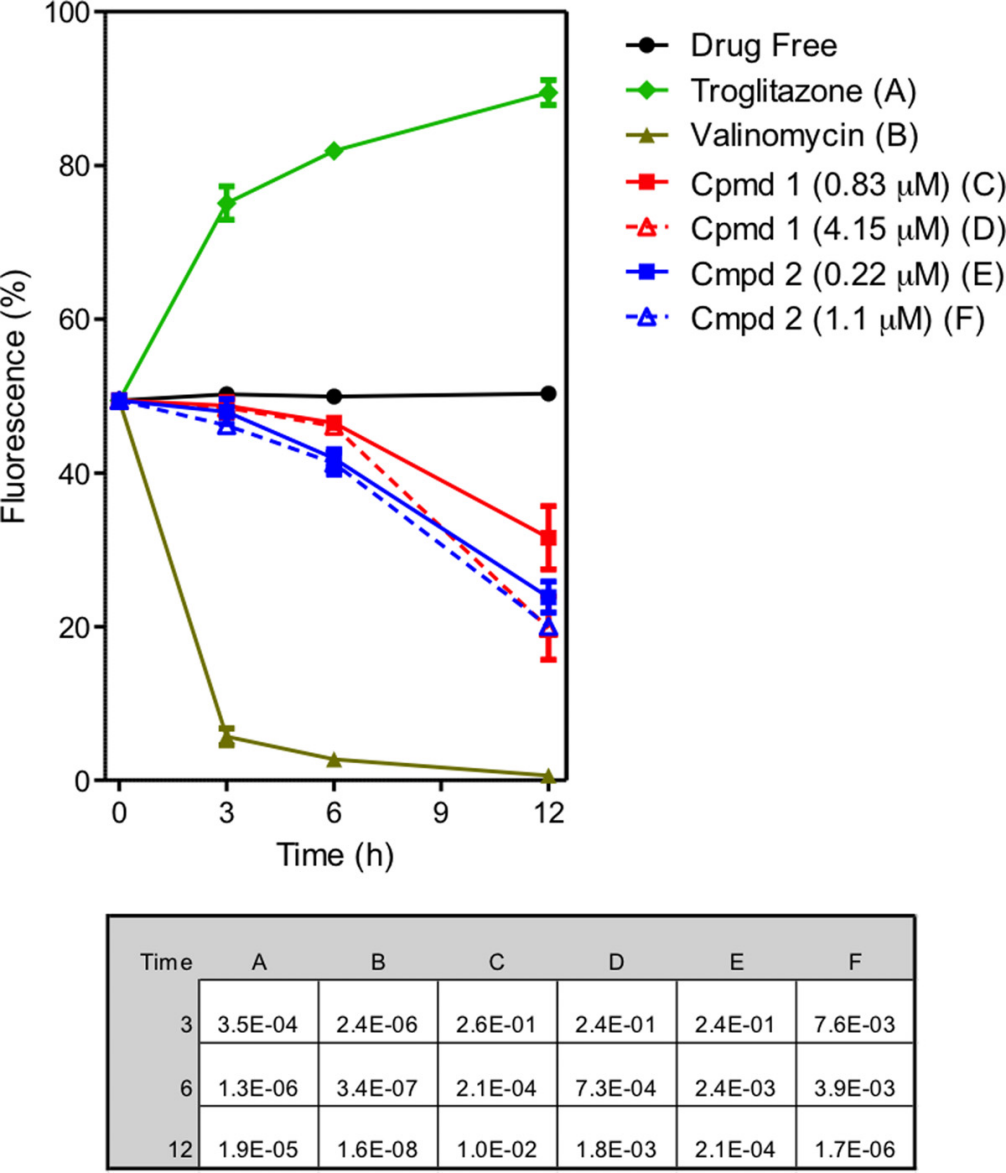
Effect of compounds **1** and **2** on mitochondrial membrane potential (Ψ_m_) of *T. b. brucei* s427-WT. The results shown are the mean of three independent determinations; error bars depict standard errors. The inset table lists the *P* values, using a Student's two-tailed, unpaired *t*-test comparing each category to the untreated control, incubated for the same length of time; a value <0.05 is considered to be significant. Untreated cells (drug free), valinomycin (negative - depolarization) and troglitazone (positive - hyperpolarization) are employed as controls.

Bloodstream forms of *T. brucei* were cultured for up to 12 h in the presence and absence of compounds **1** and **2**, stained with TMRE and analysed by flow cytometry for analysis of Ψ_m_. Valinomycin was used as the control for depolarization and troglitazone as the control for hyperpolarization. The summary of the results of three independent determinations of TMRE fluorescence of treated and untreated cells with **1** and **2** for 3, 6 and 12 h is shown as a line graph in Figure 3. Histograms of one determination are shown in Supplementary Figure S5. Values are given as the percentage of cells with fluorescence above 100 arbitrary units (A.U.), which was set to approximately 50% for the controls. A shift to higher fluorescence signifies increased Ψ_m_; lower fluorescence signifies depolarization of the mitochondrial membrane; for the untreated cell culture, Ψ_m_ remained stable for the duration of the experiment, at approximately 50%. Compounds **1** and **2** dose dependently, progressively reduced Ψ_m_, with only minor effects at 3 h but significantly progressing by 6 h, and a clear, dose-dependent depolarization of the Ψ_m_ evident at 12 h (Figure 3). The data are consistent with mitochondrial targeting of the compounds as cations, but their effect on the membrane potential is slower than has been reported for some other compounds such as bisphosphonium salts, which appeared to impact directly on the F_o_F_1_-ATPase (20).

#### Fluorescent compound **2** accumulates in the mitochondrion of trypanosomes

**Figure 4.**
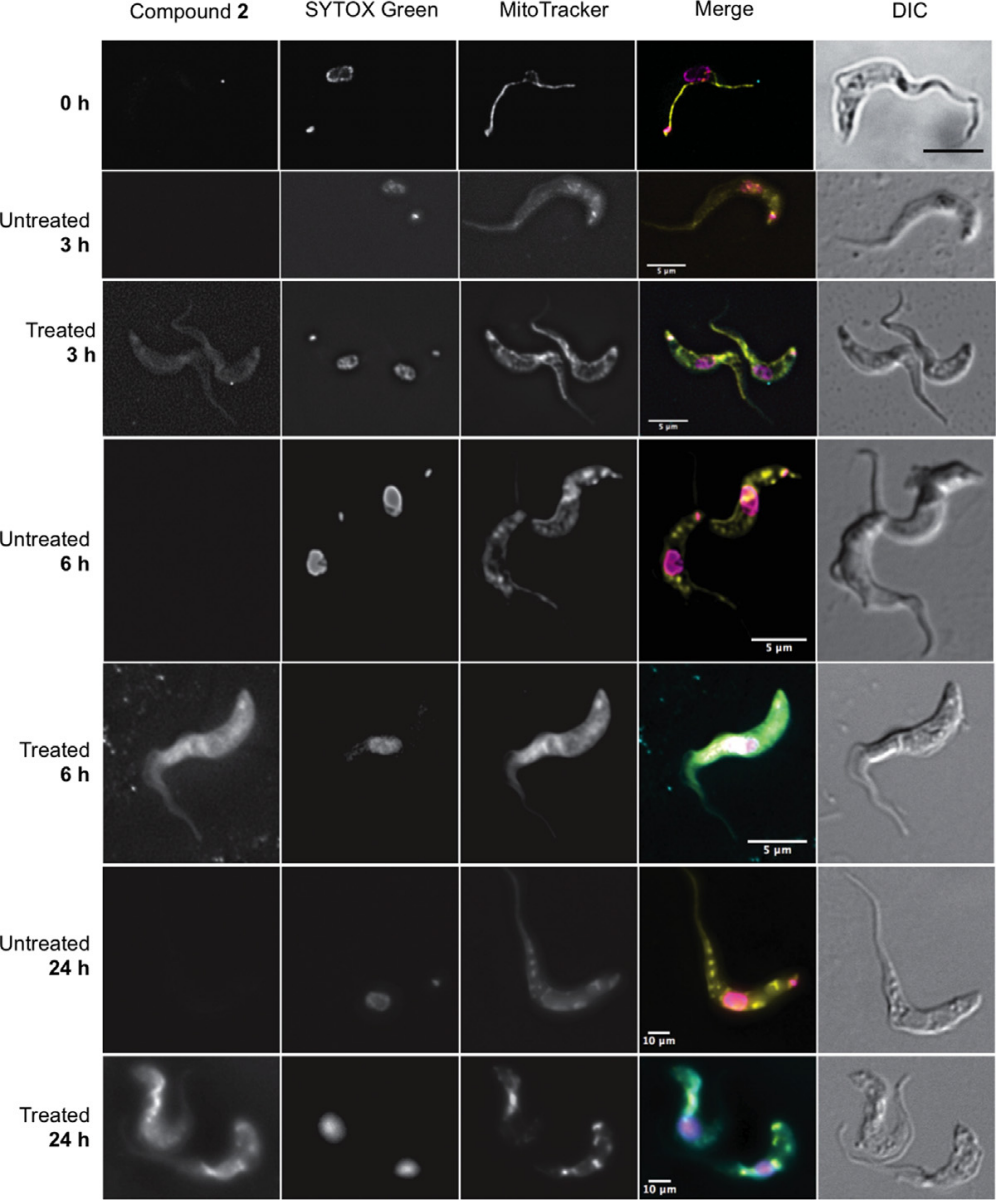
Fluorescence localization of the compound **2** in *Trypanosoma brucei* s427-WT cells. Images were taken at 3, 6 and 24 h of incubation with 5 μM compound **2**. Untreated cell samples were taken at each time point for drug free control. All fluorescent images are shown with compound **2** (λ = 450, blue channel), SYTOX Green (λ = 523, green channel), MitoTracker (λ = 599, red channel), and merge, where arbitrary colors were used to visualize the various dyes: blue for **2**, purple for SYTOX, yellow for Mitotracker. The outline of all cells is shown by differential interference contrast (DIC) imaging. Images were acquired using a DeltaVision imaging system and deconvolved using the ratio conservative method, on SoftWoRx software.

In order to study the cellular distribution of the bis(2-aminoimidazolinium) compounds, we incubated cells with the fluorescent compound **2** (emission: 450 nm), using the green-fluorescent SYTOX Green nucleic acid stain (excitation/emission: 504/523 nm) to visualise the nuclei and kinetoplasts. We further visualized the *T. brucei* mitochondrion with the red-fluorescent dye MitoTracker Red CMXRos (excitation/emission: 579/599 nm), which produces a highly specific stain of *T. brucei* mitochondria (45,46). After 3 h of exposure, compound **2** can just be detected inside of the cell but no particular distribution is discernible (Figure 4). After 6 h, compound **2** shows a homogeneous distribution throughout the cytoplasm, and is becoming concentrated in the mitochondria. At that moment, the kinetoplast is no longer visible in many of the cells, although the SYTOX Green still strongly highlights the nuclei. After 24 h of treatment, compound **2** seems to be even more concentrated in the mitochondrion with intense spots of the fluorescence coinciding with the most intense MitoTracker staining; kinetoplast staining with SYTOX was absent from almost all cells. In untreated cells grown in parallel, no changes in fluorescence were observed at the blue wavelength that visualized **2**, and there were no changes to kinetoplast DNA staining (see Figure 4).

#### Compounds **1** and **2** cause destruction of the kinetoplast DNA network

Transmission electron microscopy (TEM) was used to study the trypanosomes’ ultrastructure after exposure to 5 μM compounds **1** and **2** to analyze the effects of these compounds on the cellular structure of the parasite. Based on the information obtained with the cellular localization of the compounds in the cell by fluorescence microscopy, TEM samples were taken after 3 and 24 h incubation, as ‘early’ and ‘late’ time points to determine the structural effects of the compounds.

**Figure 5.**
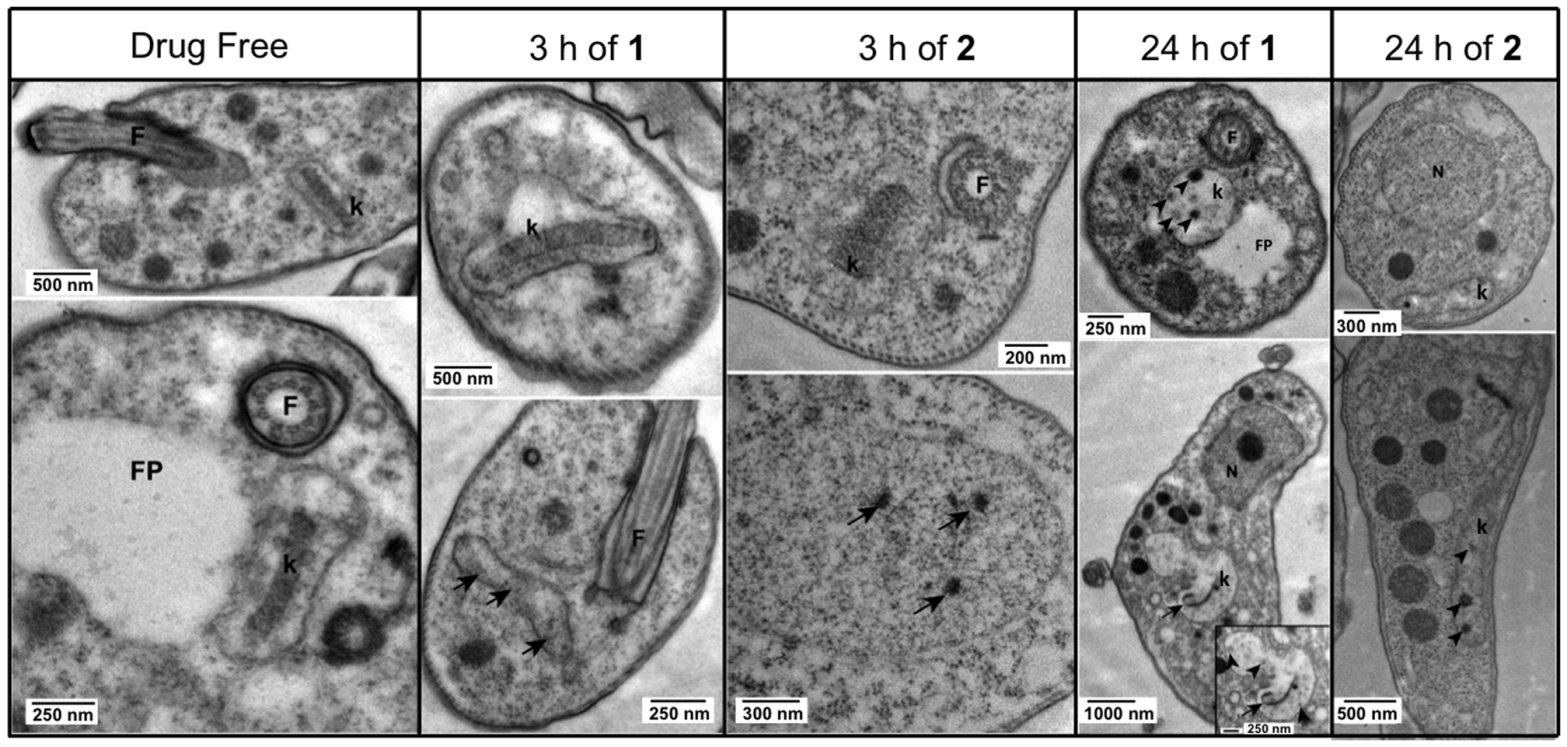
TEM images showing normal ultrastructure of bloodstream form for treated and untreated (drug free control) cells of Tb427-WT after incubation with compound **1** or compound **2** for either 3 h or 24 h. F = flagellum, FP = flagellum pocket, k = kinetoplast. Images were observed in a Tecnai T20 (FEI) at 200 kV. Irregular structures in the kinetoplast are shown with arrows and arrowheads, indicating kDNA damage.

Analyzing the images obtained with TEM (shown in Figure 5), we can confirm that compounds **1** and **2** have the same mechanism of action in the cell. Both compounds cause ultrastructural abnormalities in the kinetoplast, which were clearly far more severe at 24 h than at 3 h of incubation, when many cells appeared still undamaged, i.e. the drug has not yet impacted on the kinetoplast (top row images at 3h of **1** or **2**, Figure 5). However, different cells in the same population show dark dots or empty space inside the kinetoplast (bottom row of same columns; arrows indicate the abnormalities), which confirm an initial level of damage in the kDNA of some cells during the first 3 h exposure with the compounds. After 24 h of treatment with compounds **1** and **2**, all cells displayed clear damage to kinetoplasts, which has completely lost the characteristic disk-like structure of uniform electron-density, a structure of intercalating minicircles and maxicircles that is essential for both the functioning and replication of the kinetoplast (47). This is completely consistent with the lack of kDNA staining observed with SYTOX Green (see Figure 4) and we conclude that at 24 h the mitochondrial DNA is largely absent and completely disorganised. Since no other structures were found to be affected at the times the samples were taken, we conclude that they directly affect the kinetoplast.

#### Compounds **1** and **2** show curative activity in a mouse model of first stage HAT

The *in vivo* activity of **1** assessed earlier showed that this compound is 100% curative in the mouse model of acute *T. b. rhodesiense* infection by intraperitoneal (4 × 5 mg/kg/day) and oral (4 × 50 mg/kg/day) dosage (4,6). Here, we assayed the *in vivo* activity of the chloro analogue **2** against *T. b. rhodesiense* in the same mouse model. The intraperitoneal administration of **2** at 4 × 4 mg/kg/day increased the mean day of relapse of parasitemia (17.5 days) in mice although no cures were obtained. However, a 5-fold higher dosage (4 × 20 mg/kg/day i.p.) provided a 100% cure rate in this mouse model of stage 1 HAT.

### Binding analysis

#### SPR-biosensor experiments

##### Compounds **1** and **2** bind DNA at AT-rich sites

The binding affinity of compounds **1** and **2** to dsDNA containing GGAATAATCGCGATTATTCC [AATAAT_ATTATT], CGAATTCG [AATT], CATATATAT [(AT)_4_], and CGCGCGCG [(CG)_4_] sequences was determined by surface plasmon resonance (SPR)-biosensor experiments (Table 2). Compounds **1** and **2** bound selectively to AT-containing DNA versus CG-containing DNA (from >10-fold to >60-fold). Binding curves of both compounds were adjusted to a two-sites affinity model where primary binding constants were in the submicromolar range with slight selectivity toward AATT versus (AT)_4_ (1.8- to 2.5-fold) or AATAAT_ATTATT oligonucleotides (1.6- to 3-fold). With the dsDNA containing GGAATAATCGCGATTATTCC, compounds **1** and **2** displayed submicromolar primary binding constants (high affinity binding site) and a 133- and 48-times weaker secondary binding constants, respectively. In these experiments, secondary binding generally accounts for non-specific binding interaction with the DNA hairpin loop as previously reported (4).

**Table 2.** DNA binding constants determined by SPR for dsDNA containing AATAAT_ATTATT, AATT, (AT)_4_ and (CG)_4_ sequences^‡^

	*K_D_* (× 10^−6^M)^a^			
	dsDNA	dsDNA	dsDNA	dsDNA
	GG**AATAAT**CGCG**ATTATT**CC	CG**AATT**CG	C**ATATATA**T	CGCGCGCG
HMGA1a (Δ50–91)	*K*_1_ = 0.35	nd^b^	nd	nd
	*K*_2_ = 11.3			
HMGB1	*K*_1_ = 0.33	nd	nd	nd
	*K*_2_ = 5.91			
**1**	*K*_1_ = 0.56	*K*_1_ = 0.166^c,d^	*K*_1_ = 0.307^d^	> 10^d^
	*K*_2_ = 74.7	*K*_2_ = 207	*K*_2_ = 20.91	
**2**	*K*_1_ = 0.43	*K*_1_ = 0.262	*K*_1_ = 0.65	*K* = 6.85^e^
	*K*_2_ = 20.8	*K*_2_ = 230	*K*_2_ = 39	

^‡^dsDNA hairpins used in the study (the loop is underlined): 5’-biotin-GGGGAATAATCGCGATTATTCCCCAATAATCGCGATTATT [AATAAT_ATTATT], 5’-biotin-CGAATTCGTCTCCGAATTCG-3’ [AATT], 5’-biotin-CATATATATCCCCATATATATG-3’ [(AT)_4_], and 5’-biotin-CGCGCGCGTTTTCGCGCGCG-3’ [(CG)_4_].

^a^Binding constants for fitting to a two-site binding model.

^b^Not determined.

^c^Primary binding constant for fitting to a two-site binding model. The secondary binding constant (*K*_2_) corresponds to nonspecific binding to the hairpin loop (62).

^d^(10).

^e^Binding constant for fitting to a one-site binding model.

##### Compound **2** inhibits the binding of high mobility group proteins HMGA1a and HMGB1 to AT-rich DNA

**Figure 6.**
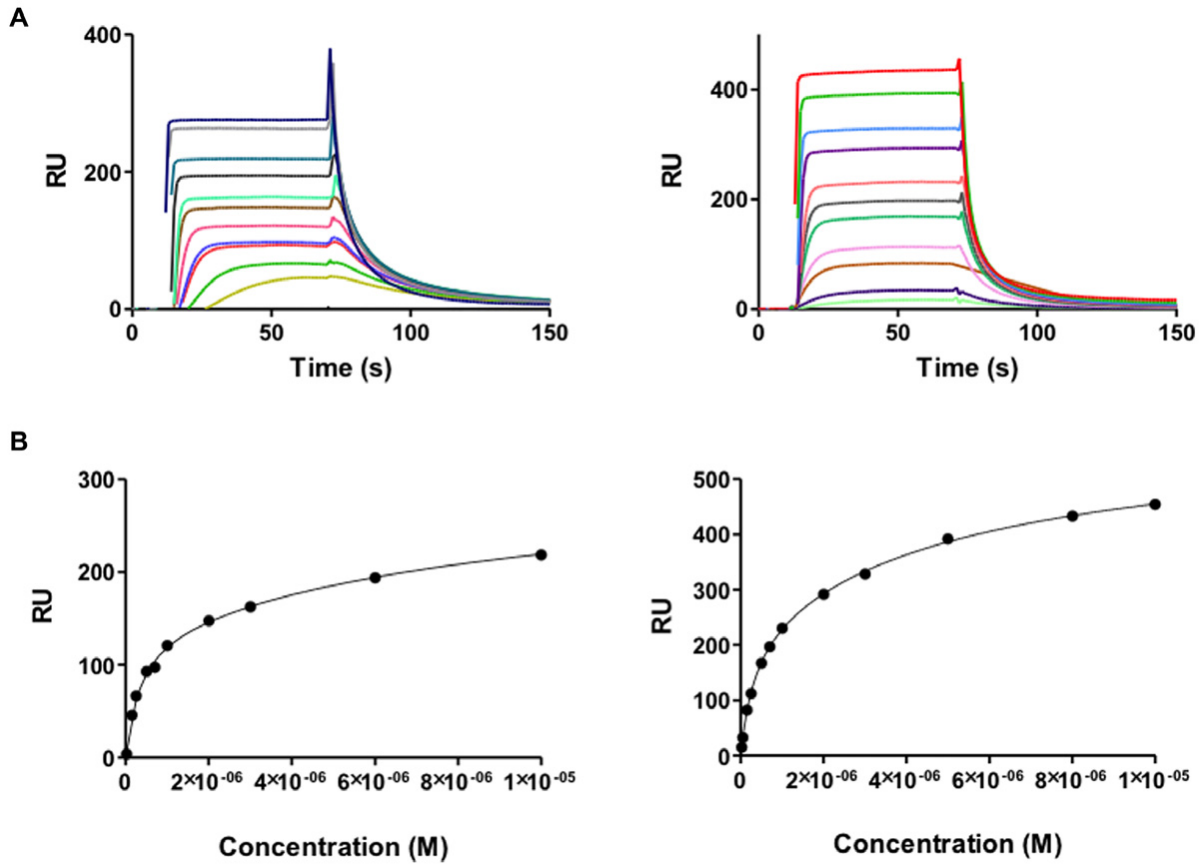
(**A**) SPR sensorgrams of HMGA1a (top left panel) and HMGB1 (top right panel) binding to dsDNA biotin-GGGGAATAATCGCGATTATTCCCCAATAATCGCGATTATT in HEPES 1 at 25°C. For HMGA1a, the best sensorgrams were obtained in HEPES 1 buffer, instead of MES or Phosphate buffer (43). (**B**) Binding curves for interaction of HMGA1a (lower left panel) and HMGB1 (lower right panel) with target DNA and fitting curve for a two-site affinity model.

The AT-rich sites of DNA are typically recognised by AT-hook proteins such as HMGA1a (48). In contrast, HMGB1 binds more non-specifically to DNA. The sensorgrams of HMGA1a and HMGB1 binding to the AATAAT_ATTATT oligonucleotide are shown in Figure 6. In our experiments, HMGA1a(Δ50–91) and HMGB1 proteins showed a high affinity binding site to the AT sequences of the AATAAT_ATTATT oligonucleotide and a weaker secondary binding site that most likely accounts for nonspecific binding to the hairpin loop (*K*_2_/*K*_1_ = 32 and 18, for HMGA1a(Δ50–91) and HMGB1, respectively). The higher affinity of HMGB1 to this nonspecific secondary binding site is compatible with this hypothesis. The *K*_D_ values and binding behavior of both proteins were similar to that of compounds **1** and **2** (Table 2).

**Figure 7.**
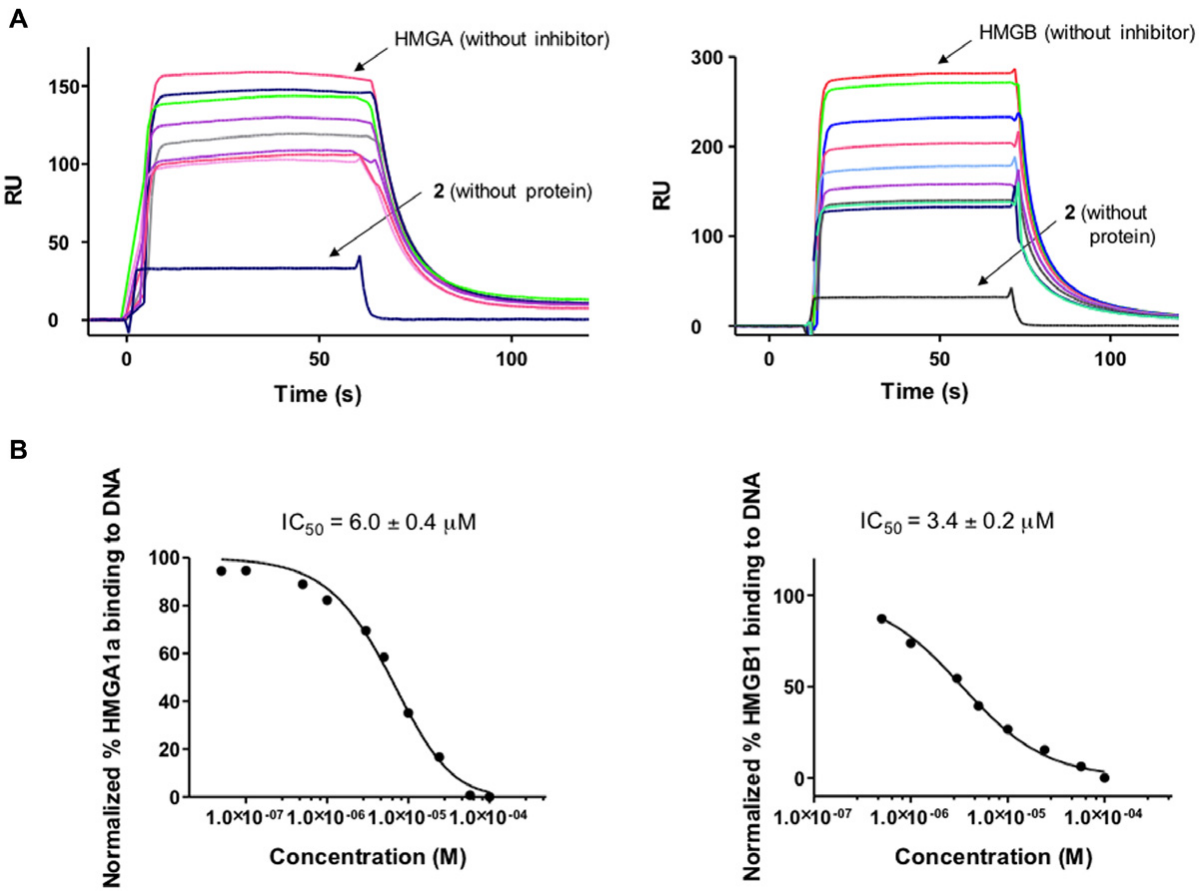
(**A**) SPR competition sensorgrams showing the inhibition of a fixed concentration (2 μM) of HMGA1a (Top left panel) and HMGB1 (top right panel) binding to dsDNA containing AATAAT_ATTATT oligonucleotide in the presence of increasing concentration of **2**. Concentration range from 0.05 to 200 μM (for HMGA1a) and 0.05 to 400 μM (for HMGB1). (**B**) Inhibition curves and IC_50_ values for inhibition of binding of HMGA1a (lower left panel) and HMGB1 (lower right panel) to dsDNA by compound **2**.

Competition assays were performed to test the capacity of **2** to inhibit the formation of HMGA1a–DNA and HMGB1–DNA complexes (Figure 7). Solutions of fixed concentration of HMGA1a(Δ50–91) or HMGB1 proteins (2 μM) in the presence of increasing concentrations of **2** were injected over the DNA immobilised surface. The sensorgrams showed a clear decrease in RU signal when increasing the concentration of **2** indicating a dose-dependent inhibition of HMGA1a and HMGB1 binding to DNA (Figure 7). IC_50_ values, determined by fitting the inhibition data with a model according to a competition system with 1:1 binding stoichiometry for HMGA1a and HMGB1 and two site-binding for competitor (43) were in the low micromolar range (6.0 and 3.4 μM for HMGA1a and HMGB1, respectively). We conclude that compound **2** competes with HMGA1a and HMGB1 binding to DNA and it is able to displace the proteins from its binding sites.

### Structural analysis

#### Structure of the DNA–compound **1** complex

**Figure 8.**
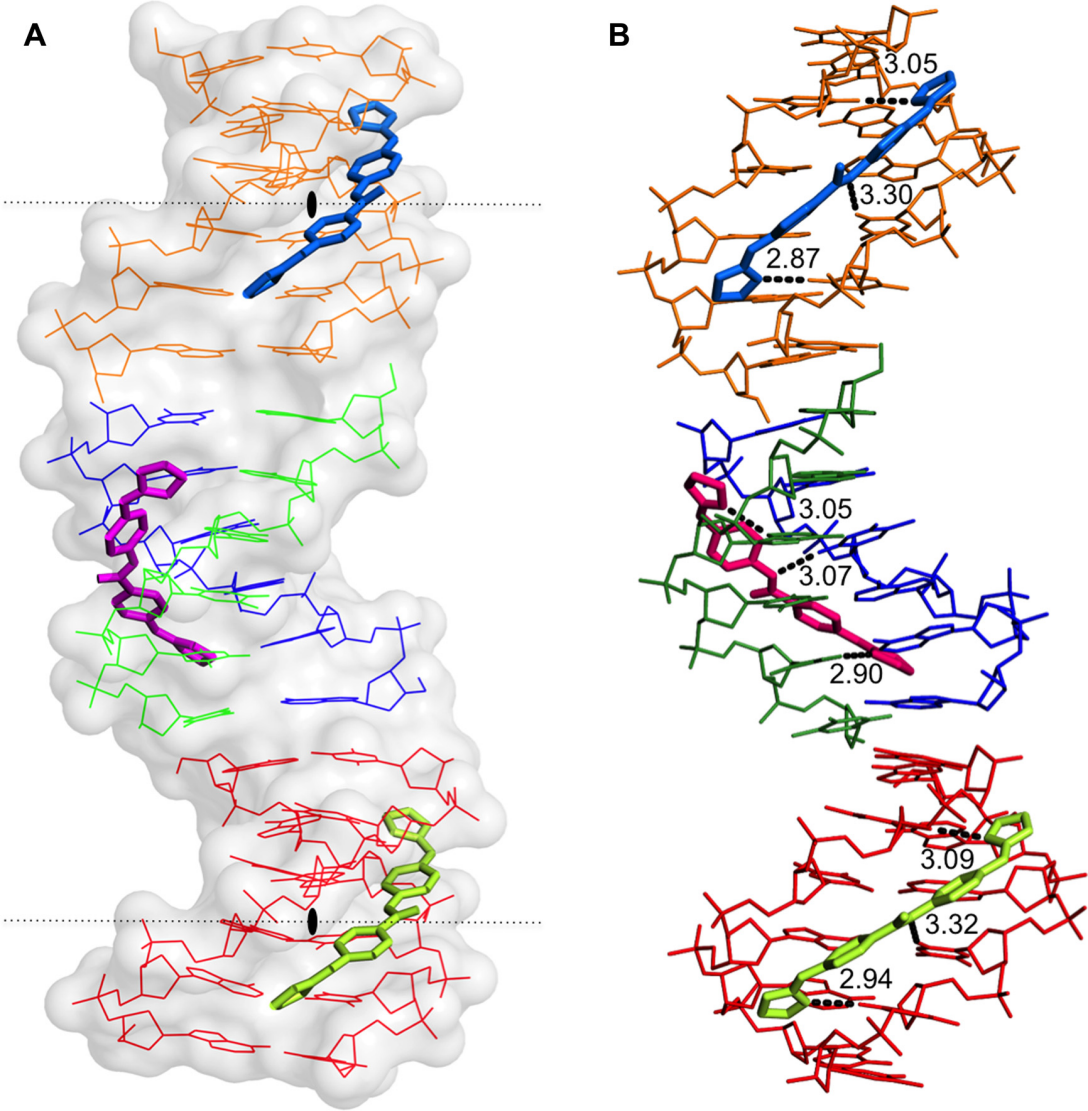
(**A**) View of the different crystallographic units of the complex. The black lozenge indicates the dyad axes. There are four independent single oligonucleotides chains; two of them (blue-green) form the central duplex and the other two (orange and red) form two different DNA duplexes with their symmetrical chain. Three crystallographically independent drug molecules are indicated in different colours. Drug F (pink), Drugs E (green) and G (blue). (**B**) Hydrogen bonds formed by the drugs with the minor-groove atoms of the DNA duplexes show similar interactions. The orientation of the aromatic rings in the central drug (F, pink) differs from to the other two drugs. Drug E and G have two possible inverted positions in the groove; for clarity only one of them is shown.

The crystal structure solved at atomic resolution of 1.25 Å of the DNA oligonucleotide [AAATTT]_2_ in complex with the compound **1** is packed in space group *I*212. A summary of crystal data and refinement statistics is given in Table 3.The asymmetric unit contains three compound **1** molecules (two of them show half-occupancy), plus one DNA duplex and two DNA single strands. Three complete duplexes and three drug molecules are shown in Figure 8. The duplexes are stacked and organised as infinite continuous parallel columns, which are packed in a pseudo-tetragonal way (Supplementary Figure S6). The drug molecules fill the central part of the minor groove of the duplexes. The crystal is stabilized in part by the interaction of the central molecule, drug F (pink) with the DNA phosphates of neighboring molecules, shown in Supplementary Figure S6. Drug G (blue) and E (green) do not participate in the interactions with neighbor DNA duplexes.

**Table 3.** Data collection and refinement statistics

Data collection	
Beamline	BL13-XALOC, ALBA
Wavelength	0.997949
Resolution range (Å)	36.14–1.25 (1.279–1.25)
Space group	*I*121
Unit cell parameters (Å, °)	*a* = 22.33, *b* = 40.25, *c* = 72.42,α = 90.00 β = 93.67 γ = 90.00
Total reflections	245 821 (22 828)
Unique reflections	16 801 (1594)
Multiplicity	14.7 (14.2)
Completeness (%)	92.54 (90.67)
Mean I/sigma(I)	14.1 (5.5)
Wilson B-factor	9.90
R-merge ^†^	0.069 (0.227)
**Refinement statistics**	
Reflections used in refinement	15 741 (1122)
Reflections used for *R*-free	850 (89)
*R*-work ^‡^	0.1205 (0.141)
*R*-free ^§^	0.1668 (0.200)
Number of non-hydrogen atoms	730
DNA	480
Compound **1**	81
Water	165
Mg^2+^	4
RMS (bonds)	0.022
RMS (angles)	2.52
Average *B*-factor, all atoms (Å^2^)	13.0
DNA	10.43
Compound **1**	10.33
Water	22.38
PDB-ID	5LIT

Statistics for the highest-resolution shell are shown in parentheses.

^†^ *R*_merge_ *=* Σ*_hkl_* Σ*_i_ |I_i_(hkl) - (I_i_(hkl))| /* Σ*_hkl_* Σ*_i_ I_i_(hkl)*.

^‡^ *R*_work_ and R_free_ were calculated as *R* = Σ*_hkl_ |* l*F_obs_*l - l*F_calc_*l */* Σ*_hkl_* l*F_obs_*l.

^§^ *R*_free_ is the *R* factor evaluated for the reflections (5%) used for cross-validation during refinement.

#### Drug conformation and interactions

**Figure 9.**
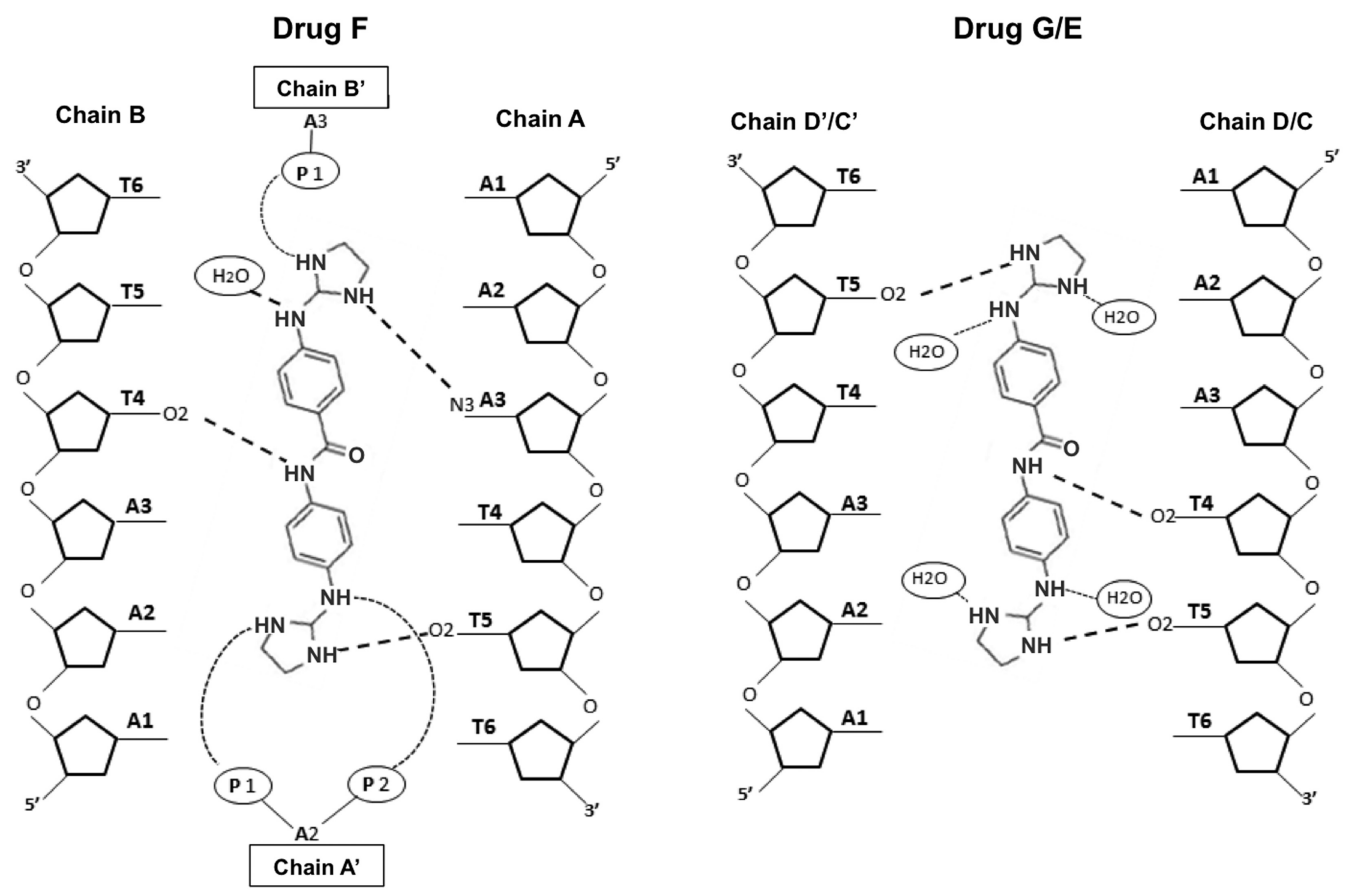
Schematic representation of interactions between F and G or E drugs and d[AAATTT]_2_. In the crystal, drugs G and E may be found in two alternative positions, up and down, which are structurally identical. Symmetric chains are indicated with apostrophe.

The asymmetric unit contains one molecule of drug F, one half-occupancy drug E and one half-occupancy drug G. All of them interact in the middle of the DNA duplex, the central AATT sequence, as found in several other cases (49). Drug E and G are practically identical (r.m.s. differences of 0.172 Å). Both drugs form three hydrogen bonds: with two consecutive thymines from one DNA strand (T4 and T5) and another thymine 5 from its symmetric strand, as shown in Figures 8 and 9. Values are given in Supplementary Table S1, available as Supplementary data. The nitrogen atoms that are not hydrogen bonded to DNA are associated with water molecules, as shown in Figure 9. The central drug molecule F has maximum r.m.s. differences of 1.250 and 1.269 Å with drug E and G, respectively. Drug F forms also three hydrogen bonds with DNA: adenine 3 and thymine 5, both from chain A, and T4 from chain B. Moreover, the drug molecules are well positioned and centered in their corresponding duplex, allowing that the central nitrogen atom (N3) interacts with the oxygen from thymine 4 [O2(T4)] either in the 3’-5’ direction (drug F with chain B) or in the 5’-3’ direction (drugs G and E) (Figure 9). A remarkable feature of this drug is its interactions with two phosphates from neighboring DNA molecules, as shown in Supplementary Figure S6 and Table S1. These cross-linking interactions contribute to stabilise the crystal lattice.

#### DNA structure

The three different DNA duplexes in the structure have similar and standard conformational parameters. The main difference is found in the twist angles in the pseudo base step T-A between the ends of neighbor duplexes in oligonucleotide columns, see Figure 8. As found in previous studies (50) the twist angle value is negative (-21°) at the contacts between oligos A-B and D-D’. Nevertheless, a peculiar feature of the present structure is the positive twist angle (29°) between oligos A-B and C-C’, allowing these two oligonucleotides form a pseudo-continuous B-DNA helix. These differences are probably due to packing constraints induced by interactions between neighboring duplexes, see Supplementary Figure S6.

#### Predicted structural changes of the crystal with compound **2**

In order to analyse the interactions of the chloro derivative **2** (Figure 1) with DNA in our solved crystal structure, the hydrogen atom in *ortho* to the right-hand imidazoline ring was replaced with a chlorine atom. The compound **2** could adopt the equivalent place as compound **1** in the minor groove of the DNA duplex. Unexpectedly, the position of the chlorine atom in compound **2** prevents the interaction with the neighbouring DNA molecules (Supplementary Figure S7), and therefore, the crystal packing would probably change to a C2 space group.

## DISCUSSION

In kinetoplastid parasites the mitochondrial DNA is organised as a tightly packed disk of maxicircles (up to ∼23 kb) with thousands of minicircles of approximately 1 kb (51). It has been argued that, because dyskinetoplastic trypanosome species exist and dyskinetoplastic strains can be selected for under laboratory conditions, the kinetoplast may not be a valid target for drugs against trypanosomiasis. Yet, although the *T. b. brucei* mitochondrion is relatively stripped-down in its long-slender bloodstream form compared to other life-cycle stages, it retains essential functions and some essential proteins, including the A6 subunit of the F_o_F_1_ ATPase, which are encoded by kDNA (52). It has now been shown that survival of the loss of kDNA requires a compensatory mutation in the nuclearly-encoded γ-subunit of the same ATPase (53). This showed that, although adaptation to the loss of kDNA is possible in trypanosomes, it requires a rare event, and that any drug causing the rapid destruction or disorganization of the mitochondrial genome, such as homidium or isometamidium (54), can be highly effective. Trypanocidal dications such as diamidines and bisimidazolinium compounds have long been speculated to act on mitochondrial targets (21,55) and some clearly bind to DNA (8,17,56). However, although their abilities to act as minor groove binders are well established, it is much less clear whether this is necessarily the principal trypanocidal mode of action, and whether binding to kDNA and/or nuclear DNA contributes most to cell death. Here, we present the most comprehensive analysis to date that one class of aromatic dications, the bis(2-aminoimidazolines), directly act on the kinetoplast and cause its rapid disintegration.

We have paid special attention to the kinetoplast since significant resistance (>100-fold) by the dyskinetoplastic cell line ISMR1, relative to the wild type cell line, indicated the kinetoplast as a probable target of compounds **1** and **2**. Moreover, a study of the *T. brucei* cell cycle by flow cytometry showed that incubation with the test compounds caused a progressive cell cycle arrest in G1 phase, reflected in the major decrease in S-phase cells after 24 h. The failure to initiate DNA synthesis was almost certainly the result of damage to the kinetoplast, as kinetoplast division necessarily precedes nuclear division (57), and both fluorescence microscopy and TEM showed major damage to the kinetoplast but not to the nucleus. Starting at just 8 h, a significant numbers of cells were found without an observable kinetoplast or with 1 kinetoplast and two or even three nuclei - clear evidence of a defect in kinetoplast replication. By 24 hours this was leading to the gross destruction and/or disorganisation of the kinetoplast as observed by TEM and concomitant growth arrest; importantly, the electron microscopy did not reveal any damage to the nuclei or other cellular structures. Despite the absence of ultrastructural damage to the mitochondrion, the membrane potential Ψ_m_ began to decrease from 6 h of incubation. In bloodstream trypanosomes, which lack the common oxidative phosphorylation complexes, Ψ_m_ is generated by the F_o_F_1_ ATPase pumping protons from the mitochondrial matrix, using ATP (53,58,59); clearly this process would progressively be impeded as the lack of functional kDNA precludes the production of the A6 subunit of F_o_, leading to the slow membrane depolarisation observed. Accumulation of cationic compounds in the mitochondria, driven by the negative electrostatic potential (negative inside), is a well-described phenomenon for cationic and dicationic compounds (18,20,60), but the relatively slow effect of compounds **1** and **2** on the Ψ_m_ compared with that of compounds that act directly on the F_1_F_0_-ATPase complex (20) is consistent with the hypothesis that the antiparasitic effect derives from preventing the accurate replication of kDNA and consequently the assembly of F_0_.

The interaction of **1** and **2** directly with DNA was confirmed by structural and binding analyses of the drug-DNA complex at the molecular level. The study of the crystal structure of compound **1** in complex with the all-AT DNA sequence d[AAATTT]_2_ at atomic resolution of 1.25 Å revealed important features (structure shown in Figure 8; electronic map is displayed in Supplementary Figure S8); The compound covers the minor groove of the DNA duplexes stacked in columns, with one compound per duplex filling the central part of the minor groove (AATT). Compound **1** may interact with DNA in several alternative positions (molecule F, G and E), which confirms its affinity for the common kDNA sequence AATT. Furthermore, we report a new mode of interaction and packing of a minor groove binding drug with DNA due to the space group I212 and the formation of hydrogen bonds with neighbouring DNA molecules. Such interactions help to stabilize the DNA structure of the stacked duplexes, and may be particularly interesting in the highly organised, densely packed kinetoplast of intercalating minicircles and maxicircles, preventing the correct separation and unwinding, that is required for the correct replication of each of thousands of minicircles (47). Further confirmation that **1** and **2** are strong DNA binders and specifically target AT- over GC-rich sites was obtained using SPR. These experiments confirmed binding constants in the low micromolar range, which are easily achieved by mitochondrial accumulation of cationic compounds (20,61).

The formation of a DNA-drug complex may disrupt the function of kDNA-binding proteins in trypanosomes, such as HMG-box proteins, topoisomerases, etc. As mentioned in the Introduction, the TbKAP6 protein has two HMG-boxes (Supplementary Figure S9) essential for kDNA replication and maintenance (23). We therefore investigated whether the bis(2-aminoimidazolinium) compounds could potentially compete with HMG-box proteins for binding to DNA. We found that compound **2** is able to displace HMGB1 (and also HMGA1a) proteins from their DNA binding sites. This could potentially be the first event in the process leading to the malfunctioning of cell cycle progression and the disruption of the kDNA organisation. Indeed, all the evidence accumulated in this work shows that compounds **1** and **2** target kDNA, leading to the destruction of the kDNA arrangement and growth arrest. As such, the prevention of HMG proteins binding to kDNA could very well contribute to their observed antitrypanosomal effect alongside the likely disruptive effect that the strong binding of the compounds to AT-rich kDNA could also have on kDNA replication.

Altogether, these results show that the *N*-phenylbenzamide bis(2-aminoimidazolinium) compounds **1** and **2** share the same mechanism of action against *Trypanosoma brucei*, acting specifically on the integrity of the kinetoplast by altering the structure and replication of kDNA. As both compounds show 100% curative activity in a mouse model of *T. b. rhodesiense* infection, they have potential as effective chemotherapeutic agents for the treatment of sleeping sickness.

## DATA ACCESS

Atomic coordinates and structure factors for the reported crystal structures have been deposited with the Protein Data bank under accession number **5LIT**.

## Supplementary Material

Supplementary Data
