# Supplementary material for: Functional and structural analysis of AT-specific minor groove binders that disrupt DNA–protein interactions and cause disintegration of the Trypanosoma brucei kinetoplast
Source: Nucleic Acids Res. 2017 Jun 16;45(14):8378–91. doi: 10.1093/nar/gkx521 (PMC5737332; doi:10.1093/nar/gkx521)
Supplement: Supplementary Data [file gkx521_supp.pdf]

SUPPLEMENTARY DATA

Functional and structural analysis of AT-specific minor groove binders that disrupt DNA-protein interactions and cause disintegration of the *Trypanosoma brucei* kinetoplast

Cinthia R. Millan, Francisco J. Acosta-Reyes, Laura Lagartera, Godwin U. Ebiloma, Leandro Lemgruber, J. Jonathan Nué Martínez, Núria Saperas, Christophe Dardonville, Harry P. de Koning, and J. Lourdes Campos

This file contains nine supplementary figures and one supplementary table.

Trypanosoma brucei brucei strain Lister 427 (Tb427WT) minicircle, complete sequence; kinetoplast [1]

LOCUS: KF293288 1001 bp DNA circular

GGGGTTGGTGTAATACACACAGGGTTTTCCCGTAGAAATTATATTAATTTGGATCATTTGGTG
TTTTCTATTGATAAAAGAATAAGATAATAGATAGATTAATTGATATTATATAGATATTATATATA
AGACGCATATAAGTGAGTCTATATACAGATAATGATGATAATTTATATATATGTTAACTTTAAT
ATTTATTTATTATTTTCTTTTCTATATTAGGAGAAATGTGATAATAGATAAGTAATGAGAGTAAT
TTAGATATTTAATTGTATATAATTACACACACAGATACGTGATATATAGAGTGTTAAGATAATA
TGATGTATATATATGTAAATTAAAAACTATTTATTATTTTTATGTTAAGTAGATGGAGAAATAAT
AGTTAAATAAGAGGTAGTACTTTGAGGAGGTATAAGGTAATATTAACATTGAGAATCTTAGAT
AACTGATAAAATACTGTTATTTCTGCATCTAAAAGAGGGTTTTAAGCTGTCTAAAAGGGTAA
AATGAGGTAATAGATAAGGTATAGATAATATAATATTTAATATAATATATATAATAACAATAGC
AGGTAAAGGTAAGAAAGTGAAGATATCATATAAGATTGTATATTTAATGTTAAACTATATTTA
TTATTTTATTTATAATTAGTAGATAAGATTAGTAGAAGTGAAGTAGTAATTGTAAAACTGATAG
TAAGATGGGAATAAGGTGTGAGATATAAATAGAAAGGTTAAGTTAATTGTAGTTATAATTGGA
AGTGCAGAAAGTGTTGTAGATGGAGTATTAGGTTGATTAGAGAGAGAGTAGTATAAAGTGTA
AAAAGTTTGTGTTGGATGGTAGAGATAGAAGGGAGAAGTTAGAAATTCAGAGAAAATTGGG
GAAAAATCAGGGAAAATCGGGCTGAAAAACCGAAAATCTTATGGGCGTGCAGATTTCACCA
TACACAAATCACGTGCTATTTTCGGGGGTTTTTAGGTCCGAGGTACTTCGAAA.

Figure S1. The **AT content** represents the **73.6%** of the minicircle genome of Tb427WT (**A = 394** and **T = 342**); and **CG-content** the **26.5%** (**G = 206** and **C = 59**). The sequence **AAA** appears **28** times and the sequence **TTT 26** times; both are shown in red, bold font. The sequence **AATT** exists **14** times and is highlighted in yellow. Tracts with more than 10 **A** or **T** are found 15 times (red and underlined).

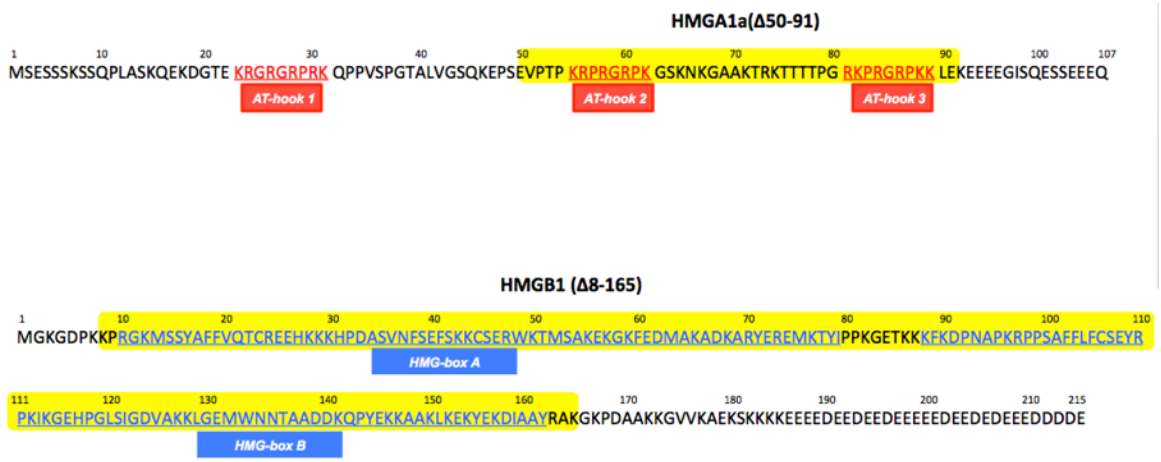

Figure S2. Full sequences of the HMG human proteins HMGA1a (top) and HMGB1 (bottom). AT-hook domains are indicated in red and HMG-box domains in blue. In both cases, the fragments used in our study [HMGA1a(Δ50-91) and HMGB (Δ7-164)] are highlighted in yellow.

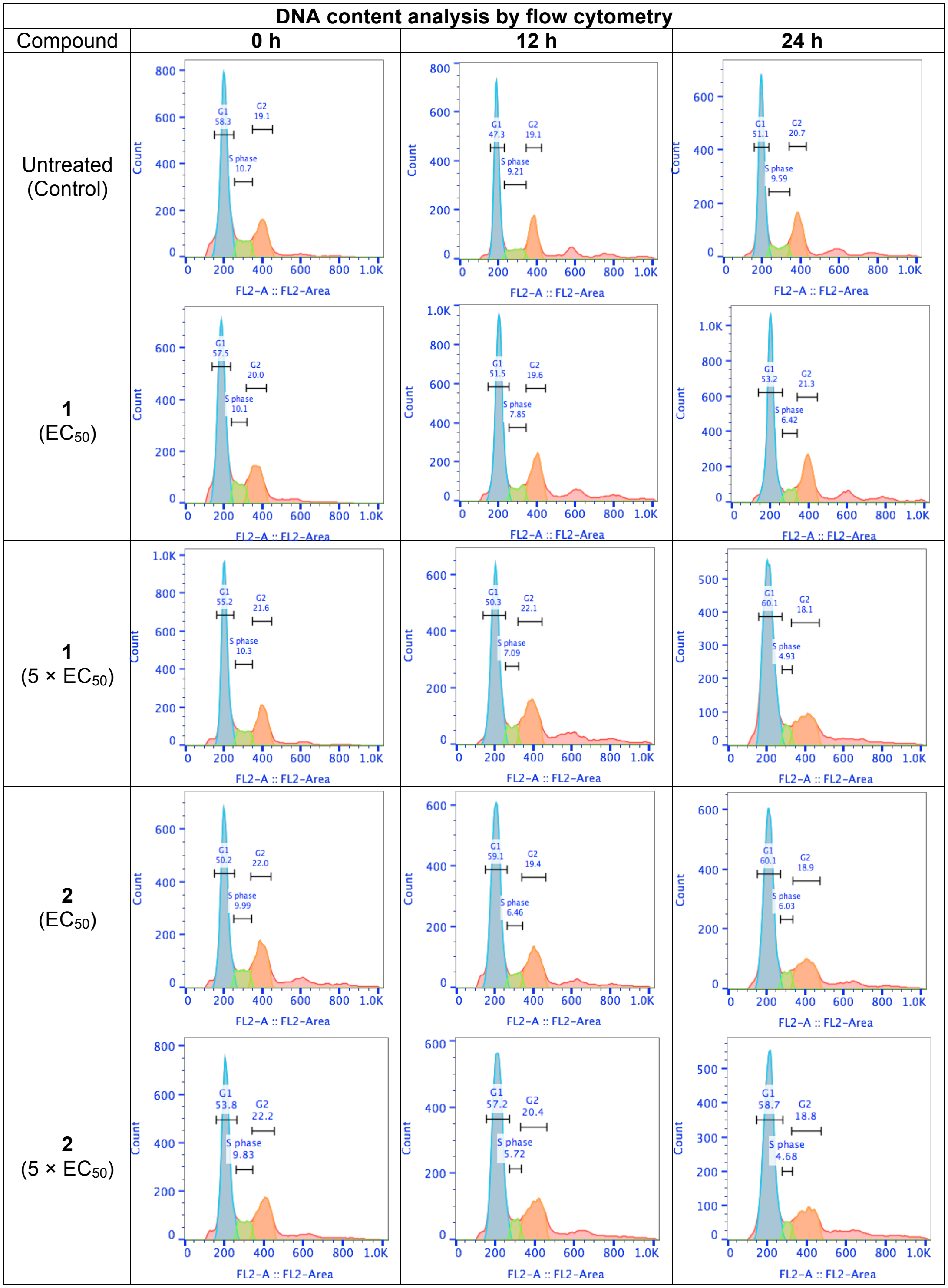

Figure S3. Flow cytometry analysis of the DNA content of bloodstream-form *T. b. brucei* s427 WT untreated and treated with the bisimidazolinium diphenyl compounds **1** and **2**. Results from one of three independent experiments that produced similar results are shown. Percentage of the population at each cell cycle phase is shown above its appropriate histogram peak. G1: all cells have one kinetoplast and one nucleus; G2: all cells have two kinetoplast and two nucleus; S phase: DNA synthesis. Untreated cells as control were included in each assay.

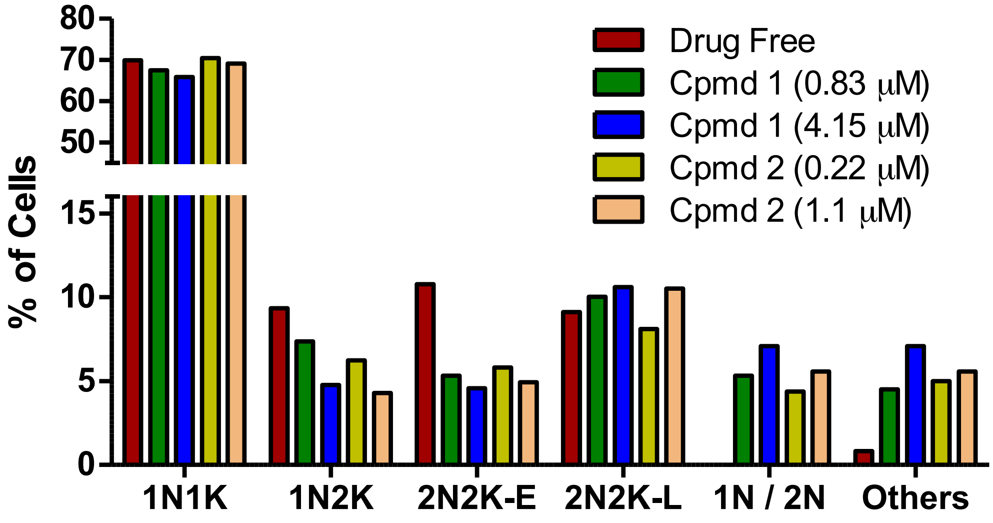

Figure S4. DNA content of cells treated 8 h with compounds 1 and 2 at 1× and 5× EC_{50} as determined by fluorescence microscopy (N, nucleus; K, kinetoplast; 1N/2N, cells with one or two nuclei but no observable kinetoplastid.

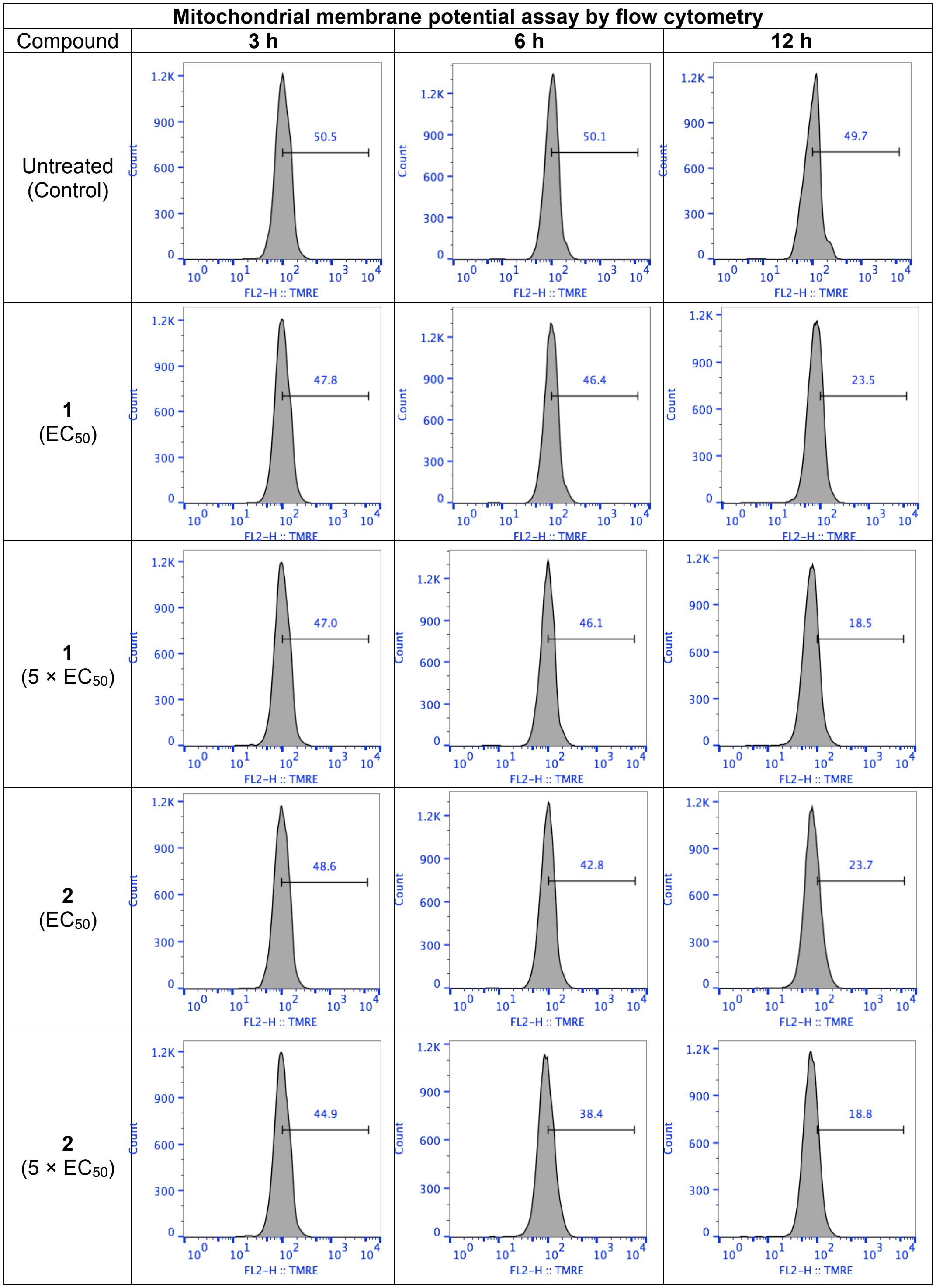

Figure S5. Histograms of TMRE-associated fluorescence after incubation with or without compounds **1** and **2**. One of three independent determinations is shown. Values are given as the percentage of cells with fluorescence above 1×10^2 arbitrary units (AU), equivalent to approximately 50% for the untreated cells. Samples are taken by a BD FACSCalibur™ using the FL2-height detector. Data were processed with CellQuest™ and ©FlowJo software.

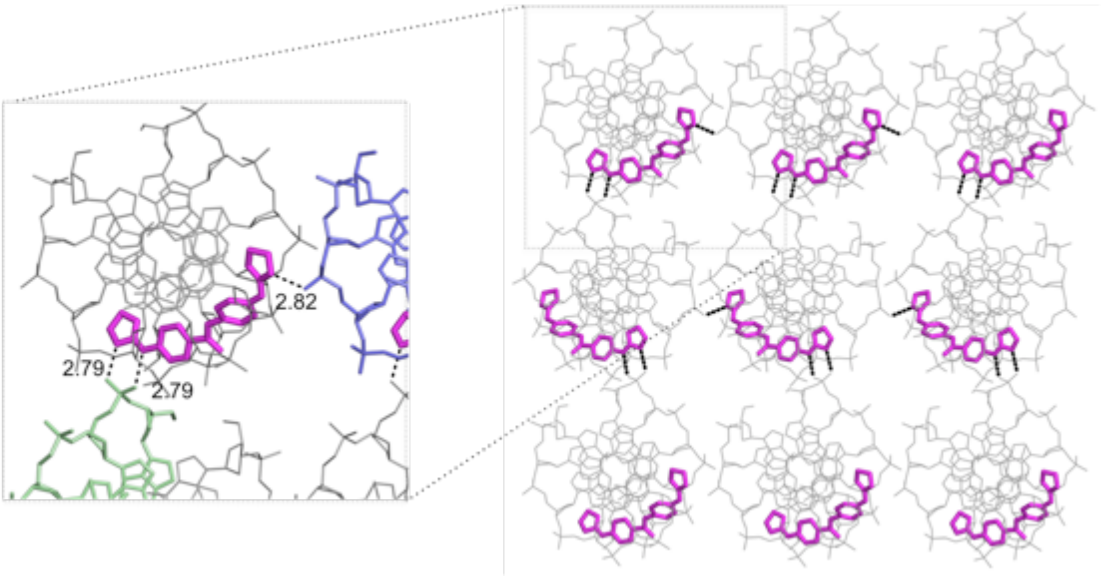

Figure S6. Packing of a layer of A-B duplexes. An enlarged view of the interactions of drug F with the neighbouring phosphates of symmetrical DNA chains is shown at the left.

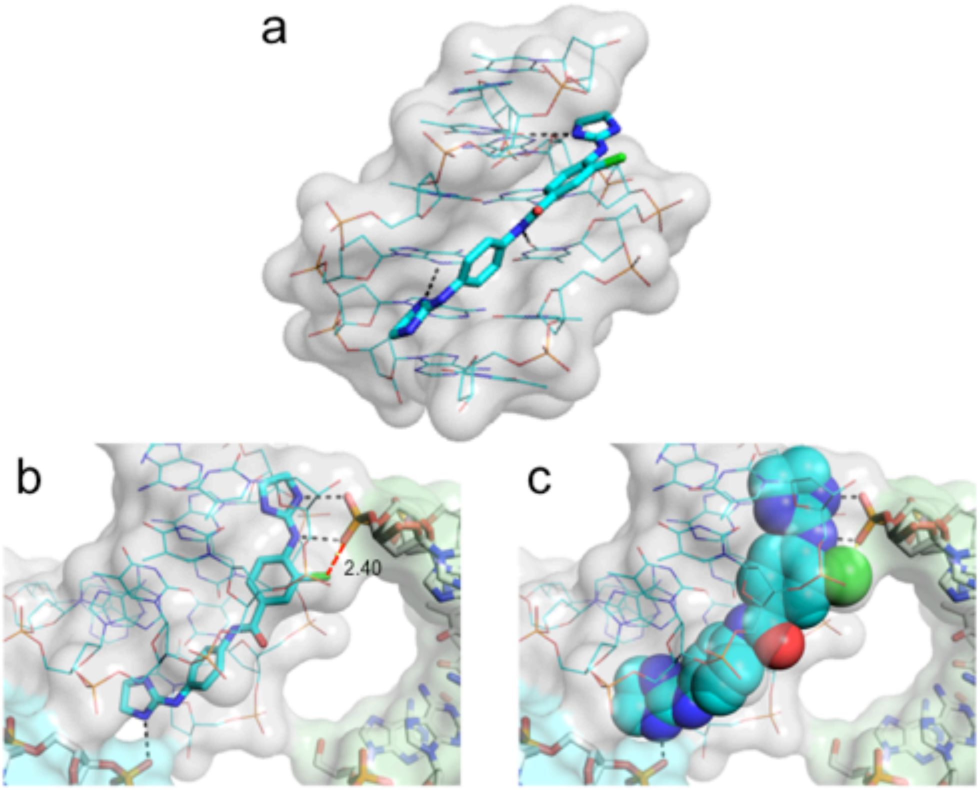

Figure S7. Structural effect of adding chlorine to compound 1. (**a**) It is clear that chlorine can be added to compound 1 without any direct influence on the interaction with DNA. (**b**, **c**) However the chlorine atom alters the interaction with the phosphate of neighboring DNA molecule, since the 2.4 Å distance is too short [red dotted line in (**b**)]. Chlorine atom is shown in green.

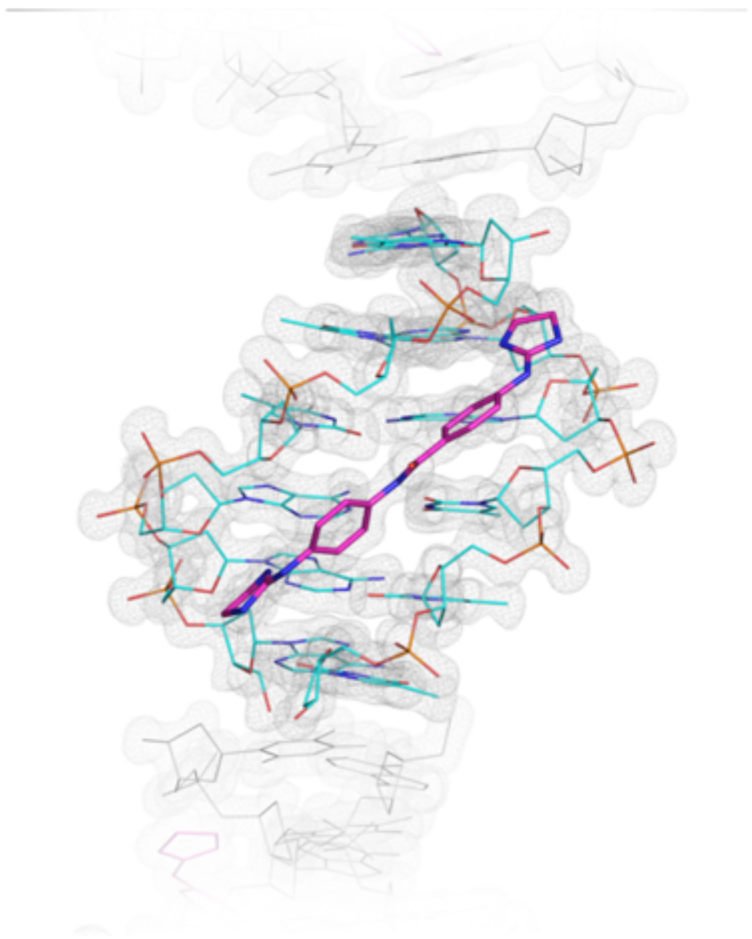

Figure S8. Electron density map ($2F_o$-F_c at 1s level) of the compound **1** drug F, in the minor groove of the all-AT DNA duplex.

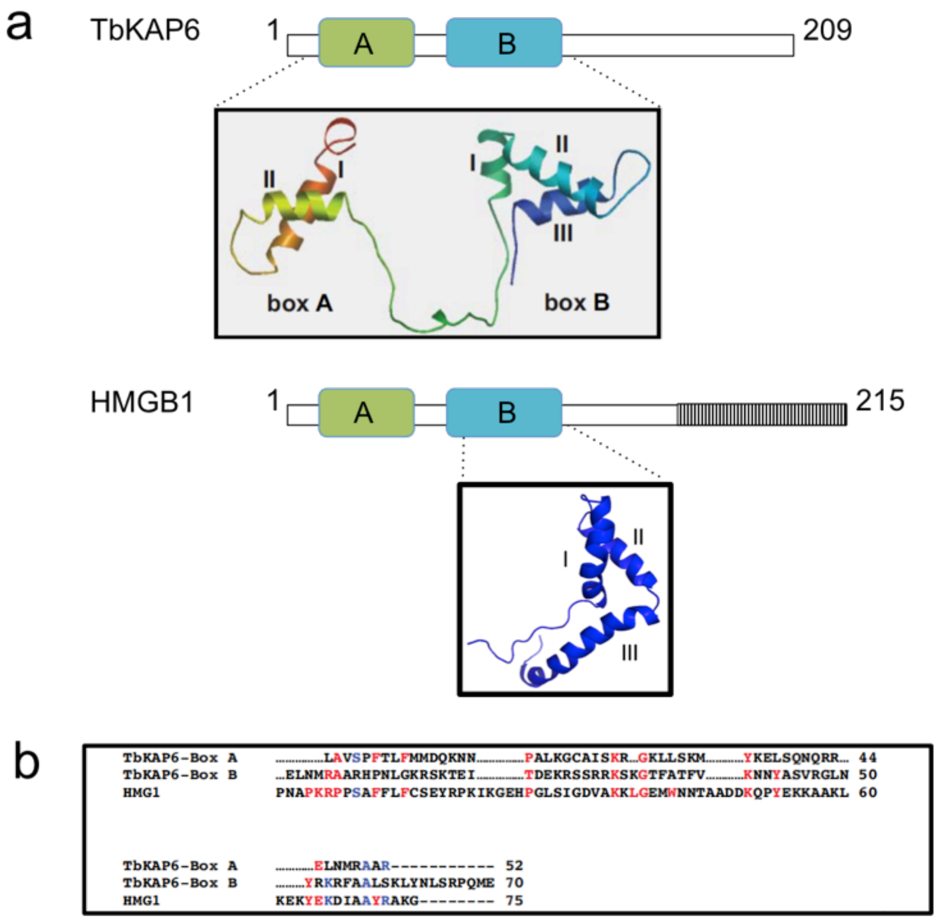

Figure S9. The HMG box-containing proteins HMGB1 and TbKAP6. **(a)** Schematic comparison of two tandem HMG-boxes in TbKAP6 with those of human HMGB1. HMG-box A: green box; HMG-box B: blue box; C-terminal acidic tails: hatched lines. Structure comparison (bellow each diagram) of the HMG boxes A and B of TbKAP6 constructed by SWISS-MODEL workspace (http://swissmodel.expasy.org/) and the HMG-box B (blue; PDB ID: 1HME) as determined by NMR microscopy [2]. **(b)** Alignment of the two HMG boxes of TbKAP6 and the human HMGB1 box B (as HMG-1). Figure adapted from Wang et al., 2014 [3].

Table S1. Hydrogen bonds formed by compound **1** in the minor groove of d[AAATTT]$_2$ and interactions with neighbouring phosphates.

Atoms involved	Drug E	Drug F	Drug G
O2(T4)-N3(6XV)	3.32	3.07	3.30
O2(T5)-N22(6XV)	--	2.9	--
N3(A3)-N13(6XV)	--	3.05	--
O2(T5)-N25(6XV)	2.94	--	2.87
O2(T5')-N13(6XV)	3.09	--	3.05
N10(6XV)-OP1(A3 oligo B')	--	2.82	--
N20(6XV)-OP2(A2 oligo A')	--	2.79	--
N25(6XV)-OP1(A2 oligo A')	--	2.79	--

Values are given in Å.

REFERENCES

1. Dean,S., Gould,M.K., Dewar,C.E., and Schnaufer,A.C. (2013) Single point mutations in ATP synthase compensate for mitochondrial genome loss in trypanosomes. *Proc. Natl. Acad. Sci. U.S.A.*, **110**(36), 14741–14746.
2. Weir,H.M., Kraulis,P.J., Hill,C.S., Raine,A.R., Laue,E.D., and Thomas,J.O. (1993) Structure of the HMG box motif in the B-domain of HMG1. *EMBO J.*, **12**, 1311–1319.
3. Wang,J., Pappas-Brown,V., Englund,P.T. and Jensen,R.E. (2014) TbKAP6, a Mitochondrial HMG Box-Containing Protein in *Trypanosoma brucei*, Is the First Trypanosomatid Kinetoplast-Associated Protein Essential for Kinetoplast DNA Replication and Maintenance. *Eukaryot. Cell*, **13**, 919–932.
